# Knowledge, attitudes, and perceptions towards waterpipe tobacco smoking amongst college or university students: a systematic review

**DOI:** 10.1186/s12889-019-6680-x

**Published:** 2019-04-27

**Authors:** Adam Arshad, Jaideep Matharoo, Ebrahim Arshad, Simardeep Singh Sadhra, Rosemary Norton-Wangford, Mohammed Jawad

**Affiliations:** 1 Medical School, College of Medical and Dental Sciences, University of Birmingham, Edgbaston, Birmingham, B15 2TT UK; 2 University Hospitals of North Midlands, Stoke on Trent, UK; 3 Public Health Policy Evaluation Unit, School of Public Health, Imperial College, London, UK

**Keywords:** Waterpipe, Public health, University students, Knowledge, Attitudes, Perceptions, Tobacco

## Abstract

**Background:**

Despite evidence for the harms of waterpipe tobacco smoking (WTS), its use is increasing amongst college and university students worldwide. This systematic review aims to assess the knowledge of, attitudes towards and perceptions of WTS among college or university students.

**Methods:**

We electronically searched MEDLINE, EMBASE, CINAHL, PSYCHINFO and ISI the Web of Science in October 2018, restricting our search to studies published since January 1990. We included studies among university or college students that used qualitative or quantitative methods, and addressed either knowledge, attitudes, or perceptions towards WTS. We excluded studies where WTS could not be distinguished from other forms of tobacco use and studies reported as abstracts where the full text could not be identified. Data were synthesised qualitatively and analysed data by region (global north/ south), and by reasons for use, knowledge of health hazards, how knowledge influences use, perceptions towards dependence, and policy knowledge.

**Results:**

Eighty-six studies were included; 45 from the global north and 41 from the global south. Socio-cultural and peer influences were major contributing factors that encouraged students to initiate WTS. Furthermore, WTS dependence had two components: psychological and social. This was compounded by the general perception that WTS is a less harmful, less addictive and more sociable alternative to cigarette smoking. Knowledge of WTS harms failed to correlate with a reduced risk of WTS use, and some students reported symptoms of WTS addiction. A large proportion of students believed that quitting WTS was easy, yet few were able to do so successfully. Finally, students believed current public health campaigns to educate on WTS harms were inadequate and, particularly in the global north, were not required.

**Conclusion:**

Reasons for WTS amongst university students are multi-faceted. Overall, interventions at both the individual and community level, but also policy measures to portray a message of increased harm amongst students, are required. Additional studies are necessitated to understand temporal changes in students’ beliefs, thus allowing for better targeted interventions.

**Electronic supplementary material:**

The online version of this article (10.1186/s12889-019-6680-x) contains supplementary material, which is available to authorized users.

## Background

In the last two and a half decades, waterpipe tobacco smoking (WTS) has spread as a recreational activity [1, 2]. Its origins date back to the late sixteenth century, when its use in mainstream society was first documented within the Middle East. Recently, its prevalence has increased in multiple settings worldwide [2–6].

The waterpipe functions as charcoal is used to heat a honey tobacco mix which then passes through a body of water before being inhaled via a long hose. The tobacco is out of sight of the user and is heated by the overlying charcoal. The air passes via the body which contains pierced aluminium foil, which helps the cooling process and prevents inhalation of charcoal ash. Subsequently, the air bubbles into the water-filled bowl and releases a mild, flavoured and fragrant vapour which is then inhaled by the user [7]. Other terms such as shisha, hookah, narghile, arghile, hubble-bubble and goza are used synonymously with waterpipe [2].

WTS has spread from Arabian to Western cultures, perhaps due to increasing globalisation and immigration, and the majority of new users are from younger age groups – particularly university students. A systematic review investigating the prevalence of WTS analysed 129 studies and showed that university students recorded among the highest of prevalence estimates worldwide (e.g. Lebanon: 65.3% ever use; Iran: 16.2% regular or occasional use) [4]. This rise in use amongst younger age groups prompted the World Health Organization to declare WTS as a growing public health concern in its 2015 advisory note [8]. This is because a plethora of published evidence highlights that WTS carries a similar risk to health as cigarette smoking [3, 9]. The constituents of waterpipe smoke, such as the presence of volatile aldehydes, polycyclic aromatic hydrocarbons and carbon monoxide, support this conclusion, since these can all lead to the development of respiratory disease and malignancy [10, 11]. Furthermore, regular use of WTS can expose an individual to high levels of nicotine and induce dependence [7, 12].

This study aims to assess the knowledge, attitudes and perceptions of WTS amongst college or university students. Given the high prevalence, reasons underlying the use of WTS in this cohort need to be explored. Previous reviews exploring this area do not clearly stratify results by college or university student status [1, 2], and it is plausible that college or university students have distinct WTS behaviours. Furthermore, the rapidly expanding literature in this field makes previous reviews out of date; a fresh review can inform the most recent discussions on policy and intervention.

## Methods

### Eligibility criteria

We included observational (cross-sectional, case-control, cohort) and interventional (randomised or non-randomised) quantitative or qualitative studies that addressed college or university students’ knowledge, attitudes and perceptions regarding WTS after 1990 for inclusion in this study. This meant that studies published after 1990, but had analysed students prior to 1990, were excluded. We defined a college or university as any educational institution with students aged greater than 18 years. If institutions had mixed samples (i.e. of university and high school students), we only included results of the university sample if available. We included and translated studies that were written in languages other than English.

We excluded studies where results of waterpipe smokers could not be distinguished from other forms of tobacco use (e.g. electronic cigarette use, cigar use), and studies reported as abstracts for which the full text could not be found.

### Search strategy

We searched five electronic databases without language restrictions in October 2018: MEDLINE (1950 onwards; access via OVID), EMBASE (1980 onwards; access via OVID), CINAHL, PSYCHINFO and ISI the Web of Science, restricting our search to studies published after January 1990.

We adapted our search strategy from strategies used in previous published literature based on the knowledge, attitudes and perceptions of waterpipe smokers [1, 2]. We also hand-searched reference lists of included studies and used PubMed’s Related Articles function. A full list of the search terms used is included in Additional file 1.

### Selection process

Two reviewers (RN and JM) independently and in duplicate screened titles and abstracts of identified citations using a standardised screening guide. Once relevant citations were selected, we obtained the corresponding full text articles. Two reviewers (AA and EA) assessed the full texts in an independent and duplicate manner using a standardised and pilot-tested screening form (Additional file 2). Disagreements regarding study eligibility were resolved by discussion or with the help of a third reviewer (SSS).

### Data abstraction

Abstracted data included:

1. Methodology: sample frame, sampling method, survey recruitment method, and survey administration method.
2. Methodological quality: sample size calculation, sampling type, validity of survey tool, pilot testing, and response rate.
3. Population: country, participant characteristics (including subject studied and socioeconomic status), setting, number of subjects in the study.
4. Outcome: knowledge, attitudes, and perceptions towards WTS.

As conducted in previous reviews on this topic [1, 2], we did not formally assess the risk of bias in each study nor stratify the analysis by study quality, as our assessment of methodological quality was considered appropriate for the broad range of study designs included.

We categorised our results by world region (global north/south), by study design (quantitative or qualitative) and then student type (i.e. general student sample, or healthcare student sample). We considered the global north to include countries from Europe, North America and Australian, and the global south to include countries from South America, Africa and Asia.

We qualitatively recorded the results according to the following themes:

1. Reasons, attitudes and beliefs that contributed to initiation and ongoing WTS.

2. Perceptions regarding the health hazards of WTS.

3. Association between knowledge and WTS use.

4. Perceptions regarding the addictive properties of WTS.

5. Perceptions regarding addictiveness compared to cigarettes.

6. Beliefs relating to WTS interventions.

## Results

**Fig. 1:**
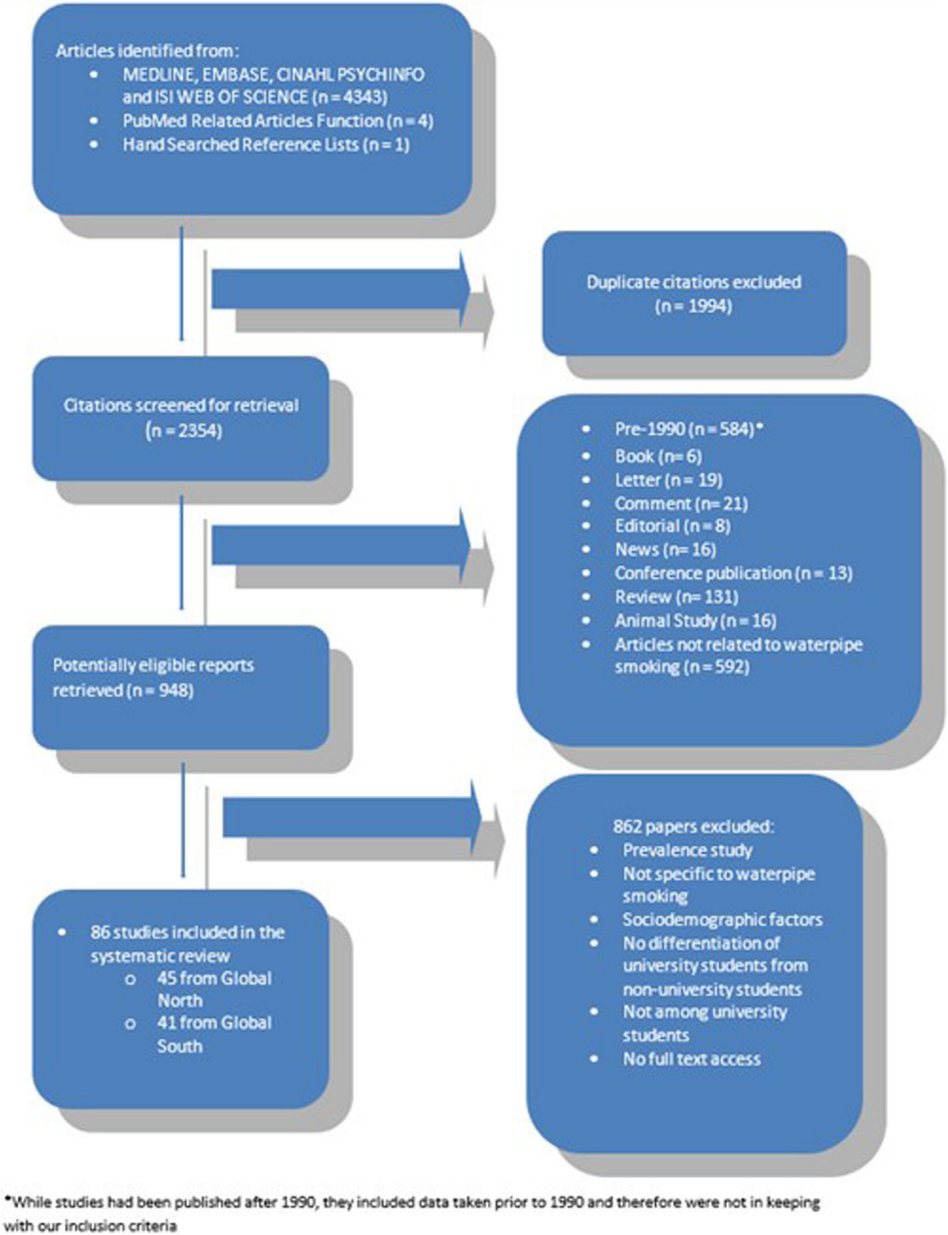
Inclusion and Exclusion of Studies included for analysis

Figure 1 shows the study flow. We identified a total of 948 papers that related to WTS and satisfied our inclusion criteria. Most of these papers (*n* = 862) were excluded for the following reasons: no knowledge, attitudes or perceptions reported, results relating to WTS could not be separated from those relating to other forms of tobacco smoking (cigarette, cigar etc.), university students could not be separated from the non-university student cohort, and no full text of the paper was available. A total of 86 papers were analysed in this review. A full list of included studies can be found in Additional files 3 and 4.

Forty-one of the 86 included studies were conducted in the global south. One study was translated from Farsi to English [13]. Eight studies were qualitative in design. Thirty-five studies were conducted via the internet, with students completing questionnaires online (4 in global south, 31 in global north).

### Reasons, attitudes and beliefs that contributed to initiation and ongoing WTS

#### The global north

A total of 16 studies assessed the reasons for initiation of WTS in the global north. Reasons for initiation of WTS included curiosity [14–18], peer/social influence [14, 15, 18–26], and wanting to overcome the social anxiety associated with new situations whilst still partaking in a legal activity [27]. Also, a positive normative belief, for example that WTS is popular amongst peers, or is a socially acceptable form of tobacco smoking that retains a certain prestige, increased the odds of initiation [16, 25, 28, 29]. This was corroborated by a quote from another study: “[it] does not appear to be hampered by the same social stigma that cigarette smoking has.” This highlights that WTS is considered to be more socially acceptable than cigarettes [16–18, 24, 30].

The most common reason associated with continued WTS was the opportunity to socialise [14, 18, 20, 21, 24, 29–37] . Other positive attitudes towards continued WTS included the belief that the practice was fun, pleasurable, attractive, and relaxing. Repeatedly, participants noted the pleasant taste and aroma of WTS as a major factor behind their use of waterpipe [15, 18, 20, 21, 29, 31, 38]. Other common reasons included: boredom, lack of other sources of entertainment, relative ease of access, habit, stress, media portrayal and the belief that WTS is less harmful than cigarette smoking [14, 18–20, 33, 39]. One study amongst American college students found that students turned to WTS as a healthier alternative to and a means of quitting cigarettes [20] . The same study found that a minority of students used waterpipe to aid weight-control, decrease appetite and improve mood and/or concentration [20]. One qualitative study amongst students who were regular waterpipe-café users in London reported that positive attitudes, such as a pleasant sensory experience, social acceptability, socializing and its use as a social lubricant, were all factors that contributed to their use of waterpipe. These students described WTS a social addiction, rather than a physiological one [18].

Finally, one cross-sectional study explored the motives behind waterpipe use amongst Muslim American college students. 54.8% of students who had ever smoked waterpipe did not rate any particular factor as being important with regards to their waterpipe use. Among the remaining students, highest-rated reasons for waterpipe use included having a good time with friends (24.7%) and being safer than cigarettes (20.5%). “Part of my culture” was rated as very important by only 4.2% of the cohort. This rating did not differ between ethnicity of the participant. Lifetime WTS was also strongly associated with the perception that most or all of undergraduate students engage in WTS (OR = 3.60). Only 26.1% of Muslim students believed that WTS was prohibited in Islam; however, believing that WTS was prohibited in Islam was not a protective factor against lifetime waterpipe use (OR 0.68, 95% CI 0.33–1.41) [33].

#### The global south

Seventeen studies assessed the reasons for initiation of WTS amongst university students in the global south [13, 40–55] . Motives for waterpipe use were similar to those reported in the global north, including sociability [13, 40, 42, 45, 47, 48, 51, 52], relaxation/fun [13, 41, 43, 44, 46, 47, 49, 50, 52], peer pressure [41, 43, 44, 50, 54, 55], curiosity [40, 44] and style [40, 45, 50, 54] . However, meeting cultural expectations and family influence – including family approval and introduction to the practice by a family member – were some of the commonest features underlying WTS cited by participants, which did not appear frequently in studies from the global north [41, 44, 46, 48, 50, 53–56] . Female students in some Arab countries cited social acceptability and tolerance towards WTS as their main motivation for use, as female cigarette smoking was highly frowned upon by Arab society [44, 45, 50, 57] . One qualitative study from Iran reported that WTS enabled women to feel empowered: “we think that it’s prestigious… one feels great, you know. Grownups do this; so you like to say – hey, I have grown up too” [51]. This was corroborated in a cross-sectional study of female Egyptian university students, whose motives for WTS included: pleasure, curiosity, and also the ability to be free to make their own life decisions [40]. Likewise, male Muslims in the Middle East described using waterpipe as the culturally and religiously acceptable form of tobacco smoking [58].

### Perceptions regarding the health hazards of WTS

#### The global north

A total of 26 studies analysed students’ knowledge, and perceptions of the health hazards of WTS [14, 18, 20, 21, 24, 26, 29, 30, 32, 33, 35–39, 59–69] . Twelve of these studies demonstrated that the majority of university students worldwide could identify some of the health hazards associated with active (rather than second-hand) WTS. These included cardiovascular disease, respiratory disease and cancer [14, 16, 18, 21, 24, 26, 27, 31, 38, 60, 69, 70]. However, students stated they were not bothered by second-hand waterpipe smoke and they would be willing to spend up to 30–60 min in the same vicinity as waterpipe smoker [20, 24] .

Current waterpipe smokers demonstrated a reduced knowledge of the harms of WTS and positive perceptions of WTS in comparison to non-users. The water filtration of the tobacco toxins, the lower temperature of the waterpipe smoke compared to cigarette smoke, and aromatic smells and pleasant taste were all cited as contributing factors to its perceived safety [18, 38].

When comparing WTS to cigarette smoking, the majority of students in the global north identified WTS as less harmful than cigarettes [18, 20, 24, 30, 32, 33, 35–37, 59, 61, 63, 65, 66, 68], whereas nine studies reported that the majority of students perceived WTS as more or equally as harmful [25, 26, 29, 39, 62, 64, 67, 69, 71].

#### The global south

Thirty studies analysed the perceptions of global south students towards WTS hazards [40–42, 44, 46, 47, 49, 50, 53–55, 57, 58, 72–88]. Sixteen studies reported that the majority of students (> 50% of the sampled cohorts) were able to identify health hazards associated with WTS [40, 41, 44, 46, 50, 53–55, 72, 75, 81, 83–86, 88]. Less than half of students in two studies reported having knowledge of WTS harms [42, 73] and in seven studies, students considered WTS to be safer than cigarettes [40, 44, 50, 75, 79, 83, 84]. Three cross-sectional studies showed that healthcare students had a greater awareness of WTS hazards compared with non-healthcare students [44, 46, 78]. In one study, 68.9% of medical students correctly identified waterpipe smoke as having significant tobacco content compared to 36.1% of non-medical students (OR: 0.3, 95% CI: 0.2–0.5) [44].

Reasons for the perceived safety of WTS included: water filtration of the tobacco [46, 49, 55, 57, 75, 77, 81], smoke not burning the lungs [77] and pleasant smell [57]. Nine studies found that the majority of students believed that WTS was equally or more harmful than cigarettes [45, 47, 54, 56, 58, 72, 74, 86, 89].

### Association between knowledge and WTS use

#### The global north

Seven studies in the global north assessed how knowledge of WTS influenced the likelihood of initiation [16, 25, 35, 60, 68, 69, 90]. In four studies, no significant correlation was identified between correct knowledge of WTS harms and a reduced probability of its initiation amongst non-smokers [16, 60, 69, 90] . In one longitudinal study, only students who answered “do not know” to questions regarding their knowledge of WTS, tar, nicotine and carcinogen content had a reduced risk of initiation of WTS after one year (aOR = 0.35, 95% CI = 0.14–0.90) [16].

Overall the greatest driver for WTS, despite even with knowledge of its harm, was the possibility to socialise, and the belief that WTS was normal [17, 22, 25, 27–30, 33, 35, 39, 69, 91]. Another longitudinal study reported higher odds for 1-year WTS in those who believed WTS to be socially acceptable and popular (OR = 8.07, 95% CI = 2.45–26.62), compared to those who did not have these beliefs [25].

In a further study from the United States, students who believed that WTS would allow them to have a good time with friends, that their friends would approve and that the smoke would taste pleasant were more likely to have intentions to smoke in the future. The beliefs that WTS would give them a good buzz, harm their health, cause family problems, cost money and that is safer than regular cigarette smoking did not significantly contribute to the prediction of intention [69]. Finally, students frequently stated that they had no access to up-to-date information regarding WTS harms [18, 39]. This has led to a state of disapproval and disbelief with public health campaigns regarding potential WTS harms.

#### The global south

One study from the global south looked at the relationship between knowledge of WTS harms and waterpipe use. In a sample of four universities in Jordan, the belief that cigarette smoking is more harmful than WTS was significantly associated with monthly waterpipe use (*p* < 0.001) [74].

### Perceptions regarding the addictive properties of WTS

#### The global north

Eighteen studies reported students’ perceptions of the addictive properties of WTS in the global north [15, 18, 20, 21, 24, 25, 30, 32, 35–37, 63, 67, 70, 91–94] . Nearly all of these studies demonstrated that respondents underestimated the addictive properties of WTS, with students not considering themselves dependent on waterpipe.

In one study from the United States, first year students stated that they had little or no risk of becoming an WTS addict, even when used socially (67%) or on their own (54%) [36]. Only one study, a Canadian survey of medical students, showed more than 50% (83/119 students) of the cohort reported WTS as addictive [92]. Reasons for the disbelief regarding the addictive properties of WTS were: limited/social use [15, 36, 91], limited exposure of addictive agents [63] and the belief that WTS is a transient behaviour as part of college [93] . Overall the proportion of student waterpipe users who wanted to quit was low – ranging from 14 to 48% across four studies [25, 26, 36, 67]. Three studies showed that the majority of waterpipe users felt that they were able to quit smoking at any time [18, 20, 21] . However, despite this perception of quitting being a straightforward task, students who had previously tried to quit WTS had often failed to do so [18, 94].

Some studies described symptoms of dependence when attempting to quit [37, 94], with students from one study from the UK reporting craving symptoms when attempting to stop WTS [37]. In another study, those who smoked waterpipe monthly were significantly more likely to report difficulties in quitting WTS (0.8% vs. 15.5%, *p* < 0.001), feeling annoyed when people criticised their WTS habits, others telling them to quit WTS (9.5% vs. 32.2%, p < 0.001) and feeling guilty about WTS (9.2% vs. 19.2%),compared to less than monthly users [94].

#### The global south

Sixteen studies explored the perceptions relating to WTS dependence amongst university students from the global south [46, 47, 49, 50, 53, 74–79, 84, 88, 95–97] . The majority of respondents regarded WTS as non-addictive, as a habit, and as something they could stop with ease [47, 49, 76, 84, 88, 96, 97]. Nevertheless, students were able to acknowledge the presence of addictive chemicals in waterpipe smoke and that this in part was because of the flavoured taste of the waterpipe smoke [77, 78]. As with the global north, the proportion of WTS users who wanted to quit ranged from 25 to 55% [41, 47, 50, 79, 95, 97], and students who had previously tried to quit WTS had often failed to do so [47, 95], with some students experiencing cravings [50]. In a cross-sectional study in Syria, 89.5% of students believed that they could quit WTS at any time; however, only 65.7% of students had attempted to quit WTS. The main motivational factors to quit WTS were health (91.6%) and cost (8.7%). The main challenges in quitting WTS were, friends (28.6%), addiction (17.1%) and boredom (8.6%), while 37.1% reported no challenges to quitting WTS [88] .

### Perceptions regarding addictiveness compared to cigarettes

#### The global north

Eight studies explored the perceived addictiveness of WTS compared to cigarettes in the global north [20, 24, 25, 29, 32, 38, 65, 71]. Six studies demonstrated that students generally perceived WTS to be less addictive than cigarette smoking [20, 24, 32, 38, 65, 71], with a higher proportion of waterpipe smokers holding this view when compared to non-waterpipe smokers [24, 38, 71].

In one study from the United States, 78.4% students who had previously smoked waterpipe perceived it to be less addictive than cigarettes. However only 44.3% of non-users believed this to be the case (adjusted OR: 3.26) [20]. In another study from the United States, 58.9% of the sample perceived WTS to be less addictive than cigarette. Students who perceived WTS to be less addictive than cigarette smoking were more likely to have smoked waterpipe in the past three months (32% vs. 11%, *p* < 0.001) [71].

#### The global south

Eight studies explored the perceptions of university students in the global south regarding the addictive properties of WTS compared to cigarettes. Across four universities in Jordan, the majority of students believed cigarettes to be more addictive than waterpipe (54.6%), with only 13.2% claiming that WTS was more addictive, and 32.2% believing that the addictive potential is about the same [74]. Similar results were reported in Bahrain [47].

In a sample of 200 Malaysian medical students, 66% considered WTS to be less addictive than cigarette smoking [75]. Similar findings were found in a sample of 645 Turkish university students [76]. In a study of 450 university students across four institutions in Pakistan, 78.8% of waterpipe smokers perceived cigarettes to be more addictive than waterpipe. However, only 62.2% of non-waterpipe smokers considered cigarette smoking to have greater addictive properties [44]. In Rawalpindi, Pakistan, 73% believed WTS to be less addictive than cigarettes. In this study, 89 participants (43%) said they felt cravings for WTS [50]. In a study of 587 university students in Syria, 89.5% of those who smoked waterpipe perceived cigarettes to have increased addictive properties compared to WTS. On the other hand, 77.1% of non-smokers believed cigarettes to have increased addictiveness when compared to waterpipe [45] .

### Beliefs relating to WTS interventions

#### The global north

Five studies explored the beliefs of students towards the interventions relating to WTS in the global north [14, 18, 39, 65, 94] . In general, students were quite dismissive of policies employed to reduce waterpipe use, stating that they had no access to public health campaigns, or that current public health campaigns surrounding WTS were not very good [18, 39, 94]. However, upon receiving information about WTS, students reported a greater worry about their waterpipe use [14, 65]. In one study from the United States, ever waterpipe smokers indicated that they were very motivated to quit smoking as a result of a health warning on packaging. According to ever users, the best location for noticing a health warning label was on the waterpipe device (41.2%), followed by the stem (36.6%) [65].

#### The global south

Five studies reported the beliefs of students towards WTS interventions in the global south. In a study of 416 students in Beirut, Lebanon, although the majority of students supported banning WTS in the workplace (81.3%), most did not agree with banning WTS use in public gardens (46.2%), among minors (age < 18 years) (14.9%), in restaurants (28.8%) and in advertising commercials (33.2%) [46]. In a separate analysis of 570 medical students in Syria, 91.7% of waterpipe smokers and 91.1% of non-smokers believed that WTS should be banned in public places [58]. The majority of individuals (74%) in a study of 228 health sciences students in South Africa believed that the practice should be subject to legal regulation [56] . A separate South African study reported that almost 50% of users believed that the dangers of WTS were exaggerated by current public health campaigns [49]. Finally, in Israel, students considered there to be a lack of awareness and knowledge of WTS [57].

## Discussion

Our findings show that the main reasons for WTS initiation worldwide were peer pressure, curiosity and socialising. Furthermore, cultural expression was another motive identified amongst Middle Eastern students in the global south. Students believed that WTS was fashionable, socially acceptable and an alternative to drinking. Furthermore, in Middle Eastern countries, WTS was more acceptable than cigarette smoking for women. Knowledge of the health risks of WTS did not deter its use among students in the global north. Meanwhile, several studies in the global south concluded that students had a lack of knowledge regarding WTS harms and addictive properties. However, there was little willingness to quit, and those who tried often struggled.

Two systematic reviews have assessed knowledge, attitudes, or perceptions of waterpipe users. A 2013 review by Akl et al. found similar motives for WTS amongst adult cohorts in both the global north and south, including socializing, peer pressure and cultural identity. While adults had similar knowledge as to the health harms of WTS, in the global south, adults perceived WTS to be less harmful than cigarette smoking. This is in comparison to our findings, where most global south students reported WTS to be more harmful. WTS was considered to be less addictive than cigarette smoking and easy to quit. In another systematic review published in 2014 focused on the factors surrounding WTS use amongst young people worldwide, reasons for WTS use were similar to those reported in our study, including entertainment, relaxation, boredom and culture. Furthermore, young people perceived there to be little harm with WTS use and minimal addictive properties. However, these reviews do not conclusively compare findings between the global north and south, are not specific to the university cohort and do not discuss specific themes such as knowledge and perceptions regarding WTS policy. Specifically, it is interesting to report in our findings that while students are able to report correct knowledge of WTS harms, this may not deter them from WTS use (a finding that differs from studies of adult WTS users [2]). Furthermore, students consistently report current WTS public health methods as ineffective and particularly, in the global north, are dismissive towards new WTS policy. Finally, while students do not consider themselves addicted, symptoms of WTS dependence are reported amongst students in both the global north and south. Altogether, this highlights a worrying trend of ineffective current WTS policy, with current efforts towards curbing WTS use being unsuccessful in deterring students.

Our study has a number of strengths. To our knowledge, this is the first systematic review exploring knowledge, attitudes and perceptions specifically toward WTS amongst college or university students. We followed the PRISMA methodology to conduct this review. Our findings cover both the global north and south, and different student populations. Analysing the results according to different world regions allowed us to identify culture-specific knowledge, attitudes and perceptions.

The major limitation of this review relates to the methodological shortcomings of the included studies (e.g. the use of non-standardized tools to measure knowledge, attitudes and beliefs). In particular, longitudinal studies are useful to assess the knowledge, attitudes and beliefs towards WTS as they allow us to explore temporality. Further research using this design is warranted to strengthen our understanding of students’ perceptions and WTS habits, and to allow us to see the influence of education interventions/public health campaigns on students WTS initiation. Furthermore, a validated survey instrument for measuring knowledge, attitudes and perceptions towards WTS should be developed. This tool could be used to monitor the efficacy of interventions implemented at local or national level. Finally, our review does not distinguish between non-tobacco (so-called ‘herbal’) or tobacco waterpipe. ‘Herbal’ waterpipe is often incorrectly advertised as a healthier alternative to a tobacco waterpipe with fewer toxic components [98, 99]. It is unclear whether students’ perceptions are in fact perceptions of ‘herbal’ waterpipe, so future studies should consider specifying the type of waterpipe product smoked.

Overall, students’ knowledge, attitudes and perceptions to WTS are multifaceted. Common themes of entertainment, kinship and social activity underlie many students’ positive attitudes. Furthermore, students do not commonly identify WTS harms, and instead regard WTS as a safer alternative to cigarettes with minimal dependence risk.

A collaborative effort between healthcare professionals, universities, policy-makers and the individual, are needed to address core misconceptions amongst university students and provide education of WTS harms. While we have identified that current knowledge of WTS harms doesn’t correlate always with reduced use, improving baseline knowledge and removing the distrust amongst students is essential if we are to reduce the amount of WTS used amongst university students.

### Implications for practice

#### Individual level

In the UK, healthcare professionals working in primary care are key providers of health education to the individual. Where appropriate, GPs should enquire about a students’ WTS habits, inform them of WTS harms and aim to realign positive perceptions. Referral to NHS Stop Smoking Services should also be offered. However, it should be noted that, despite this service accommodating for waterpipe users, users report infrequent referral, suggesting that GP education is warranted to inform patients of this service [18]. In a recent questionnaire study, it was seen that GPs had lower harm perception, gave less cessation advice, and made less referrals for WTS and self tobacco users compared to cigarettes, highlighting the need for improved education amongst primary care doctors [100].

#### Community level

South Asian and Middle Eastern students cited culture as a reason for their positive perceptions of WTS. Therefore, interventions involving religious and community leaders might help decrease the prevalence of this habit. There is a general lack of evidence of effectiveness for most waterpipe interventions [101], although one study involving community leaders in rural areas of Egypt has showed promising results [102]. Furthermore, interventions should aim to target the university community collectively. Although institutions may offer tobacco cessation services, specific WTS health promotion is preferable. One method for this is a bottom-up approach that empowers students to educate one another. In previous studies, healthcare students have demonstrated a better understanding of WTS harms compared to their non-healthcare counterparts [44, 46, 78] and might provide a starting cohort for this style of intervention.

#### National level

Policy-makers must explicitly target WTS to ensure that the practice is controlled amongst students and must work to ensure that public safety and health is maintained. For example, policy-makers could approach waterpipe use by limiting the number of waterpipe bars and cafes within certain areas (e.g. a 10-mile radius from a university campus) especially as students cite ease of access as an influencer for WTS. Furthermore, there is evidence to suggest that waterpipe cafés are purposely located near educational establishments [64, 103]. They could also ensure that existing policy is upheld by clearly displaying health warnings on waterpipes and in waterpipe bars and cafes. In addition, increasing waterpipe product taxation may further deter students from smoking [104].

A high quantity of positive waterpipe messages and advertisements exist online, adding to the positive perceptions of WTS [105]. This justifies legislation regulating online waterpipe advertisement. Studies also show that social media can provide effective, cost effective health promotion to student cohorts and as such should be considered as part of wider health awareness campaigns [105, 106].

## Conclusion

Our systematic review identifies that college and university students wrongly perceive WTS to be a non-harmful, and non-addictive form of tobacco use. Targeted public health campaigns, educating this at-risk cohort as to the adverse effects of WTS are required if we are to effectively prevent health complications from waterpipe use.

## Additional files

Additional file 1: Search Terms. Search Terms used for the identification of relevant studies. (DOCX 16 kb)

Additional file 2: Data Extraction Form. Proforma used for the extraction of data from each included study. (DOCX 16 kb)

Additional file 3: Global North Studies. Characteristics of all included Global North Studies (DOCX 133 kb)

Additional file 4: Global South Studies. Characteristics of all included Global South Studies. (DOCX 111 kb)
