# Supplementary material for: Knowledge, attitudes, and perceptions towards waterpipe tobacco smoking amongst college or university students: a systematic review
Source: BMC Public Health. 2019 Apr 27;19:439. doi: 10.1186/s12889-019-6680-x (PMC6487066; doi:10.1186/s12889-019-6680-x)
Supplement: Supplementary file 1 — Search Terms. Search Terms used for the identification of relevant studies. (DOCX 16 kb) [file 12889_2019_6680_MOESM1_ESM.docx]

Additional file 1: Search terms used for the 5 databases utilised

arkeela

argilah

argileh

argeela

arghile

arghil

argil

cachimba

Galyan

Ghalyan

Ghalyaan

Ghelyoon

gouza

goza

guza

hookah

hooka

huqqa

huqqo

hukka

hubble bubble

hubble-bubble

hubbly-bubbly

calean

kalian

Lula

Lulava

Maasel

Mu’assel

Moassel

nargileh

narghile

nargil

narghil

nargile

narguile

nargeela

Nargila

Okka

Qalyān

shisha

sheesha

 Tobamel

 waterpipe

water pipe

water-pipe

| Search terms | Medline | Embase | Web of Science | PsychInfo | CINAHL Plus (EBSCO) |
| --- | --- | --- | --- | --- | --- |
|  |  |  |  |  |  |
| 1. arkeela OR argilah OR argileh OR argeela OR arghile, arghil OR argil OR cachimba, Galyan OR Ghalyan OR Ghalyaan OR Ghelyoon OR gouza OR goza OR guza OR hookah OR hooka OR huqqa, huqqo OR hukka OR hubble bubble OR hubble-bubble OR hubbly-bubbly OR calean OR kalian OR Lula OR Lulava, Maasel OR Mu’assel OR Moassel OR nargileh OR narghile OR nargil OR narghil OR nargile OR narguile OR nargeela OR Nargila OR Okka OR Qalyān OR shisha OR sheesha OR Tobamel OR waterpipe OR water pipe OR water-pipe | 1596 | 1887 | 30486 | 355 | 503 |
