# Supplementary material for: Knowledge, attitudes, and perceptions towards waterpipe tobacco smoking amongst college or university students: a systematic review
Source: BMC Public Health. 2019 Apr 27;19:439. doi: 10.1186/s12889-019-6680-x (PMC6487066; doi:10.1186/s12889-019-6680-x)
Supplement: Supplementary file 2 — Data Extraction Form. Proforma used for the extraction of data from each included study. (DOCX 16 kb) [file 12889_2019_6680_MOESM2_ESM.docx]

Additional file **2: Screening form to identify relevant full texts**

| **Study** | **Methodology** | **Methodological qualities** | **Population and Setting** | **Results** |
| --- | --- | --- | --- | --- |
| **Global North/Global South** | | | | |
| **[Author and year]** | - Sampling frame: - Sampling Method: - Recruitment method: - Administration method: | - Sample size calculation: - Sampling type: - Validity of tool: - Pilot testing: - Response rate: | - Country: - Participants: - Setting: - N sampled: - N participated: - N analyzed: |  |
