# Supplementary material for: Knowledge, attitudes, and perceptions towards waterpipe tobacco smoking amongst college or university students: a systematic review
Source: BMC Public Health. 2019 Apr 27;19:439. doi: 10.1186/s12889-019-6680-x (PMC6487066; doi:10.1186/s12889-019-6680-x)
Supplement: Supplementary file 3 — Global North Studies. Characteristics of all included Global North Studies (DOCX 133 kb) [file 12889_2019_6680_MOESM3_ESM.docx]

| **1.**  **Young adult smoker risk perceptions of traditional cigarettes and nontraditional tobacco products.**  **Richter et al.**  **2006** | - Sampling frame: Phonebook and Campus Newspapers - Sampling Method: Convenience sample or purposive sample - Recruitment method: Phone, Internet, Mail - Administration method: In person, group discussion. | - Sample size calculation: No - Sampling type:   Non-probability sampling   - Validity of tool: Not reported - Pilot testing: Not reported - Response rate: N/A | - Country: America - Participants: Males or females either attending 2- or 4 year college, or not attending college. Mixed ethnicities. (Hispanic, Black etc.). - Setting: Not stated - N sampled: N/A - N participated: 151 - N analyzed: 151 | - WTS was rated safer than their preferred cigarette varieties by many participants. - A Hispanic in college said: "well, it doesn't really hurt when you inhale. It feels about the same feeling but (inaudible) when you're smoking cigarettes it feels like it's more harmful than shisha. - The availability or amount of the product smoked was mentioned as a reason that WTS was more less harmful than a traditional cigarette. A non-Hispanic white college student said: "I put safer (for WTS compared to cigarettes) because they say if you smoke it [Shisha] in a bong or whatever well you're not going to carry that around, so you wouldn't be smoking as much. |
| --- | --- | --- | --- | --- |
| **2.**  **Prevalence of and associations with waterpipe tobacco smoking among U.S. university students.**  **Primack et al.**  **2008** | - Sampling frame: University of Pittsburgh Students - Sampling Method: Simple Random Sampling - Recruitment method: Email - Administration method: Internet | - Sample size calculation: Yes (power calculation) - Sampling type: Probability Sampling - Validity of tool: Self developed tool, no validation reported.      - Pilot testing: Yes - Response rate: 18.6% | - Country: USA - Participants: Comparing the responders to the non-responders, respondents were younger (20.9 vs. 21.4, p<0.001), more likely to be female (65.6% vs. 50.5%, p<0.001), and more likely to be Caucasian (85.4% vs. 80.7%, p=0.004). Of the respondents, 39.9% lived on campus, and 8.5% reported being a member of a fraternity or sorority. - Setting: College - N sampled: 3,600 - N participated: 660 - N analyzed: 647 | - A third (33.1%) believed that WTS was less harmful than cigarette smoking, and over half (52.1%) believed that WTS was less addictive than cigarette smoking. - 36.4% considered WTS as “very socially acceptable.” - 1-year waterpipe smoking was associated with low perceived harm (OR=2.54, 95% CI=1.68, 3.83) and low perceived addictiveness (OR=4.64, 95% CI=3.03, 7.10). Odds ratios for 1-year waterpipe smoking were higher for those who believed its social acceptability was moderate (OR=8.07, 95% CI= 2.45, 26.62) or high (OR=20.00, 95% CI=6.03, 66.30), compared with those who believed its social acceptability was low. - Similarly, odds ratios were increased for those who believed it was moderately (OR=3.34, 95% CI=2.04, 5.50) or highly (OR=4.72, 95% CI=2.85, 7.82) popular, compared with those who believed it was not popular |
| **3.**  **Prevalence, knowledge, and practices of hookah smoking among university students, Florida, 2012.**  **Rahman et al.**  **2014** | - Sampling frame:   Data from 478  university  students from a  university situated  in Florida   - Sampling Method:   Convenient   - Recruitment method:   Not reported   - Administration method:   Self-Administered | - Sample size calculation: No - Sampling type: Non-probability sampling - Validity of tool: Self developed tool, no validation reported (but questions were based on expert and a literature review. - Pilot testing: Yes - Response rate: Not Reported | - Country: USA – Florida - Participants:   Women (54.6%) and 17  were men (45.4%)  78.8% were  ndergraduate students.  Participants  were of varied  ethnicities including  white (33.4%), Asian  21.3%), Hispanic  (17.4%), and black  12.8%).   - Setting: Large urban university in Florida. 2011 - 2012 - N sampled: N/A - N participated: N/A - N analyzed: 478 | - 30% of never users documented intention to smoke waterpipe in the future citing ‘opportunity to socialise’ as the main reason for a change in habit - 74.6% stated that there are health risks associated with WTS. 50.6% associate cigarette smoking as more dangerous than WTS. - 70.3% use their own judgement when assessing health risks associated with WTS, having no means to gain health education regarding WTS. |
| **4.**  **Relationships among factual and perceived knowledge of harms of waterpipe tobacco, perceived risk, and desire to quit among college users.**  **Lipkus et al.**  **2014** | - Sampling frame:   Newspaper  Advertisements.  Flyers posted  around 7  campuses in North  Carolina   - Sampling Method:   Convenient   - Recruitment method:   Mail   - Administration method:   Internet | - Sample size calculation:   Not reported     - Sampling type:   Non-probability sampling   - Validity of tool:   Questions on actual  knowledge of  dangers of  waterpipe were  from previously  reported validated  tool.  Perceived worry of  harm was a  idenification of  Dijkstra &  Brosschot’s 4-item  worry scale used  or cigarette  smoking.  All other questions  were self  developed,  non-validated.   - Pilot testing: Not reported - Response rate: | - Country: USA - Participants:   Summing across the  samples, the mean age  was 20.3 years (SD=2.0,  range 18 to 32); 65%  were men. In terms of  race and ethnicity, 66%  were Caucasian, 14%  Asian/Pacific Islander, 9% African-American, 7% other, and 4% Hispanic   - Setting:   October 2009 – July 2011. College.  Study 1:   - N sampled:1177 - N participated: 108 - N analyzed: 91   Study 2   - N sampled: 153 - N participated: 126 - N analyzed: 112   Study 3   - N sampled:131 - N participated: 121 - N analyzed: 113 | - Students who perceived they had good knowledge regarding WTS harms worried about illnesses if they were to continue smoking. But they were not worried about addiction. - If a student has ‘factual’ and ‘perceived’ knowledge about WTS, they have a greater perceived risk of the addictive properties of WTS. - Similarly, those who smoked cigarettes considered themselves to be more likely to become addicted to WTS than non-smokers. |
| **5.**  **Social context of smoking hookah among college students: scale development and validation.**  **Sharma et al.**  **2013** | - Sampling frame:   Local waterpipe cafes  around college  campuses   - Sampling Method:   Snowball sampling   - Recruitment method:   In person (initially,  then they  informed others)   - Administration method:   In person, interviewer administered however an in person, self-administered questionnaire was also conducted | - Sample size calculation:   No   - Sampling type:   Non-probability sampling   - Validity of tool:   Self-developed tools, no validation reported, but based on previously used tools/experts  “Items generated in  initial pool of  interviews were  reviewed by a  content expert  (tobacco control)  and the other of  whom was an  expert on  instrument  development.  They provided  feedback on the  content validity”   - Pilot testing: Yes - Response rate: In those approached, response rate 90% | - Country: USA - Participants:   64.2% males, 19%  freshmen, 27%  sophomores, 28.5%  juniors and 25.5%  seniors.  More than one-third  identified themselves as  white (43.4%) or  Asian (39.8%).   - Setting:   Participants recruited from waterpipe cafes near the campus. Setting of interviews not stated.  In-person surveys were  also conducted in the  dormitories from  students who identified   - N sampled: N/A - N participated: 274 - N analyzed: 274 | - A principle component analysis revealed 3 reliable factors for WTS use: social facilitation,   family/cultural influence, and alternative to smoking, cigarettes and drinking.   - Weekly waterpipe users were more likely to smoke in a context of social facilitation than the other 2 groups. - Similar effects were observed for family/cultural influence. |
| **6.**  **Knowledge, attitudes, and normative beliefs as predictors of hookah smoking initiation: a longitudinal study of university students.**  **Sidani et al.**  **2014** | - Sampling frame: Undergraduate and Graduate students at the University of Florida - Sampling Method   Convenient Sampling   - Recruitment method:   Email   - Administration method:   Internet | - Sample size calculation:   No   - Sampling type:   Non-probability sampling   - Validity of tool:   Self-developed tools, validation reported from a variety of sources.   - Pilot testing:   Not reported   - Response rate: 36% for baseline, 67% for follow-up (of the 852 who did baseline). | - Country: USA - Participants:   Compared to the entire  university population  respondents (n = 852)  were younger  (20.6 vs. 21.1, p  =  .04),  more commonly female than male (46.8% vs. 40.0%, p < .01), and more commonly White than non-White (71.0% vs. 58.7%, p < .001)   - Setting: University of Florida. 2010 – 2011 - N sampled: 2400 - N participated: Baseline – 852, Follow-up - 569 - N analyzed: Baseline – 852, Follow-up – 569/852 | - There was a significant association between positive attitudes and odds of initiation (OR 1.93-3.12) and negative attitudes and a decreased Odds of initiation (0.55-0.77). - No relationship is seen between correct/incorrect knowledge of WTS harms and its initiation. - However, students who answered ‘don’t know’ regarding their knowledge of WTS tar, nicotine and carcinogen content were associated with a reduced initiation risk (AOR = 0.35, 95% CI = 0.14 – 0.90, AOR = 0.12, 95% CI 0.03 – 0.5 and AOR = 0.28, 95% CI = 0.11 – 0.70). |
| **7.**  **Intention to smoke tobacco using a waterpipe among students in a southeastern U.S. College.**  **Noonan et al.**  **2011** | - Sampling frame:   Undergraduate  students at South  Eastern university   - Sampling Method:   Simple random sampling   - Recruitment method:   Email   - Administration method:   Internet | - Sample size calculation: No - Sampling type: Probability Sampling - Validity of tool: Self developed tool.   Validaton reported (uses for example, Fishbein-Ajzen-Hanson Questionnaire)   - Pilot testing: Yes - Response rate: 26% | - Country: USA - Participants:   The mean age was 19.9.  54% were female.  The majority were  Caucasian American.  61% had ever waterpipe  use and 13.5% reported  current waterpipe use   - Setting: University. Spring 2009 - N sampled: 100 - N participated: 261 - N analyzed: 223 | - Positive attitudes towards WTS and believing its use was   ‘normal’ were associated with  intention to smoke waterpipe within the next 3 months. |
| **8.**  **Knowledge and attitudes toward hookah usage among university students.**  **Holtzman et al.**  **2013** | - Sampling frame:   Students at a  university in south  Eastern USA.   - Sampling Method:   Convenience sampling   - Recruitment method:   Internet and in person   - Administration method:   Internet | - Sample size calculation:   No   - Sampling type:   Non-probability sampling   - Validity of tool:   Self-developed tool, no validation reported.   - Pilot testing:   Not reported   - Response rate:   Not reported | - Country: USA - Participants:   376 males, 533 females,  34). Mean age 20.02  (SD = 1.74). The study  sample was ethnically  diverse  (i.e, 16.3% Asian, 9.5%  African American,  13.7% Hispanic or  Latino, 59.4%  Caucasian, 1.1% other),  and was relatively  representative of the  overall student  population, which is  7.7% Asian, 8.0%  African American,  18.1% Hispanic Latino,  58.4% Caucasian, 5.0%  other.   - Setting: College. February 2009 – January 2010 - N sampled: 943 - N participated:914 - N analyzed: 914 | - Socialising, taste, relaxation, smell and boredom were the most important reasons given for smoking with ORs of 3.32, 2.58, 2.24, 2.11 and 2.01 respectively. - Many students believed WTS is equally or less   harmful/addictive than cigarettes.   - Many further believe   cigarettes contain more nicotine.   - Students were willing to spend an hour in the presence of second hand waterpipe smoke. - No relationship is noted between perception/knowledge of WTS and initiation. - 97.3% those using the waterpipe were ‘very confident’ in their ability to quit. - 78.4% students who had previously smoked waterpupe perceived it to be less addictive than cigarettes. However, only 44.3% of nonusers of waterpipe believed this to be the case (Adjusted OR 3.26). - Some students used WTS to quite cigarettes and also to lose weight. |
| **9.**  **Hookah use among college students from a Midwest University.**  **Braun et al.**  **2012** | - Sampling frame:   Registered  students at a  midwestern  university   - Sampling Method:   Simple random sampling   - Recruitment method:   Email   - Administration method: Internet | - Sample size calculation: Yes - Sampling type: Probability Sampling - Validity of tool: Self developed tool, validity reported - Pilot testing: Not reported - Response rate: Not reported | - Country: USA - Participants:   Sample consisted of 174  males (40%).  Freshmen responded  the most frequently  (30%) followed by  sophomores (23%),  juniors (24%), and  seniors (23%).  The majority of  respondents identified  themselves as  Caucasians (77%; n =  338) followed by  African Americans  (12%; n = 51) and  Asian/Pacific Islander (4%; n = 19).  12% were a member of  a social fraternity or  sorority   - Setting: Midwestern University – Date not detailed - N sampled: 2000 - N participated: N/A - N analyzed: 438 | - 98% of respondents were introduced to waterpipe through their friends - 96% primarily smoke waterpipe with friends and 29% cite ‘group influence’ (i.e. peer pressure) as their main reason for continued use - 88% (43 students) stated WTS is harmful to health. Students identified WTS as associated with: respiratory illness (92%), Cardiovascular illness (69%) and Cancer (69%) - 98% users of waterpipe were under the impression that they could quit smoking at any point in time. |
| **10.**  **A comprehensive examination of hookah smoking in college students: use patterns and contexts, social norms and attitudes, harm perception, psychological correlates and co-occurring substance use.**  **Heinz et al.**  **2013** | - Sampling frame:   Undergraduate  Introductory  psychology class at  an urban  Midwestern  university   - Sampling Method:   Convenience sample   - Recruitment method: Not reported - Administration method:   Participants completed questionnaires in groups. Unclear if participants completed questionnaires on their own or with others. | - Sample size calculation: No - Sampling type: Non-probability sampling - Validity of tool: Previously used, validated tool - Pilot testing: Not reported - Response rate: Not reported | - Country: USA - Participants: No   baseline demographics reported of participants.  Among waterpipe  users, the most  frequent age  of first waterpipe use  was between 17 and  18 years old (n = 36,  52.9%), followed by  16 (n = 19, 27.9%).  Proportion of life-time waterpipe use was highest among Caucasian (60.0%), followed by Asian and Hispanic participants (43.48% and 48.15%, respectively)   - Setting: Midwestern university. Date unknown - N sampled: N/A - N participated: N/A - N analyzed: 143 | - Half of the sample studied reported WTS to be considered more socially acceptable than cigarette smoking amongst their peers with 1/3 supporting this as their reason for continuing to smoke waterpipe. - Students believed WTS is less harmful than cigarettes. - 75% students who claimed to utilise the watepipe perceived the act of WTS to be less addictive than cigarette smoking, compared to 28% non-smokers. |
| **11.**  **A descriptive study of smoking tobacco using a waterpipe among college students.**  **Noonan et al.**  **2013** | - Sampling frame: University Students - Sampling Method: random - Recruitment method: Email - Administration method: Internet | - Sample size calculation: No - Sampling type: probability - Validity of tool: Self developed tool no validation reported - Pilot testing:No reported - Response rate: 23% n=223 | - Country:USA - Participants: University Students. Male 73%, Female 27% - Setting: University - N sampled: 1000 - N participated: 223 - N analyzed:223 | Answering the question: “If I smoke tobacco using a waterpipe… It will give me a good buzz” (M:F)   - Likely 60 (58.8%): 38 (31.4%) - Unlikely 42 (41.2%): 83 (68.6%)   I will have a good time with friends   - Likely 24 (23.5%): 60 (49.6%) - Unlikely 78 (76.5%): 61 (50.4%)   It will taste pleasant   - Likely 65 (63.7%): 37 (30.6%) - Unlikely 37 (36.3%) 84 (69.4%)   I may harm my health   - Likely 91 (89.2%): 110 (90.9%) - Unlikely 11 (10.8%) 11 (9.1%)   It is safer then cigarette smoking   - Likely 41 (40.2%): 26 (21.5%) - Unlikely 61 (59.8%): 95 (78.5%)   It will cost a lot of money   - Likely 28 (27.5%): 50 (41.3%) - Unlikely 74 (72.5%): 71 (58.7%)   “If I smoke tobacco using a waterpipe I will get lung cancer”   - Likely 63 (61.8%): 77 (63.6%) - Unlikely 39 (38.2%): 44 (36.4%)   It will smell pleasant   - Likely 66 (64.7%) 45 (37.2%) - Unlikely 36 (35.3%) 76 (62.8%)   It will help me relax   - Likely 55 (53.9%) 26 (21.5%) - Unlikely 47 (46.1%) 95 (78.5%)   It is less irritating then cigarettes   - Likely 20 (19.6%) 43 (35.3%) - Unlikely 82 (80.4%) 78 (64.5%)   Overall In this study , male participants held more positive beliefs about WTS (that it would smell pleasant, taste pleasant, would give them a good buzz and would help them relax).  More males than females thought that WTS was a safer alternative to cigarettes (41% vs. 26%) and that waterpipe smoking was less irritating than cigarette (43% vs. 20%). |
| **12. Associations between hookah tobacco smoking knowledge and hookah smoking behavior among US college students.**  **Nuzzo et al.**  **2013** | - Sampling frame: University Students 2339 - Sampling Method: Simple Random - Recruitment method: Email - Administration method:   Internet | - Sample size calculation: No - Sampling type: Non-Probability - Validity of tool: Self developed tool no validation - Pilot testing: No - Response rate: 36% | - Country: USA - Participants: 2339 - Mean age 19 Male 53% Fem 47% - Setting: University campus - N sampled: 2339 - N participated: 852 - N analyzed: 852 | - There was a substantial knowledge gap among a random sample of college students, with the vast majority of individuals unaware of the toxin load associated with waterpipe tobacco smoking. |
| **13. Beliefs and norms associated with smoking tobacco using a waterpipe among college students.**  **Noonan et al.**  **2012** | - Sampling frame: University Students N=993 - Sampling Method: Random - Recruitment method: Email - Administration method: Internet | - Sample size calculation: No - Sampling type: Non-probability - Validity of tool: Previously validated tool (TRA Waterpipe questionnaire) - Pilot testing: Not reported - Response rate: 261 (26%) | - Country: USA - Participants: University Students   Male 102 Fem 159  Mean age 20  Asian 29 Black 7 Caucasian 160 Hispanic 8 Other 19   - Setting: University - N sampled: 993 - N participated: 261 26% - N analyzed: 223 (23%) | - Believing WTS would allow them to have a “good time with friends” and would “taste pleasant” were more likely to have intentions to smoke in the future. - Believing WTS will “give me a good buzz” and that it is “safer than regular cigarette smoking” although positively associated with intentions, did not significantly contribute to the prediction of intention. - Similarly, beliefs that WTS would “harm their health” and would “cost a lot of money” were negatively correlated with smoking intentions but did not significantly contribute to the prediction equation. - Current waterpipe and current cigarette smoking significantly contributed to the prediction of smoking intentions. - Students who believed that their friends and significant others would approve of their smoking were more likely to have intentions to smoke in the future. - Perceived approval from parents and siblings although positively correlated with smoking intentions did not significantly contribute in predicting intentions. |
| **14. Differing psychosocial risk profiles of college freshmen waterpipe, cigar, and cigarette smokers.**  **Smith-Simone et al.**  **2008** | - Sampling frame: University Students - Sampling Method: Unknown - Recruitment method: Unknown - Administration method:   Internet | - Sample size calculation: Unknown - Sampling type: Unknown - Validity of tool: Unknown - Pilot testing:   Unknown   - Response rate:   Unknown | - Country: USA - Participants: Male 212, Female 199 - Setting: University - N sampled: Unknown - N participated: 411 - N analyzed:411 | - University freshmen believed the following: they were least likely to get addicted when using waterpipes socially, they were most likely to be influenced by their friends to use waterpipes in the next year, waterpipe tobacco smoking was the most socially acceptable form of tobacco smoking among their peers; and they perceived their peers to look coolest when using waterpipes. - Using cigarettes as the reference product, the following individual psychosocial risk factor differed statistically from cigarettes: lower likelihood of addiction when using cigars and waterpipes socially, higher likelihood of peer influence to use waterpipes in the next year, and perception that peers looked cooler when using cigars and waterpipes. - Based on the overall mean P3AS (Product Attractiveness Score) waterpipes had the lowest overall mean score followed by cigars, and cigarettes, indicating that peer use of waterpipes was perceived most attractive overall. |
| **15. Ethnicity and waterpipe smoking among US students.**  **Abughosh et al.**  **2012** | - Sampling frame: University Students - Sampling Method: Simple Random - Recruitment method: Email - Administration method: Internet | - Sample size calculation: Not reported - Sampling type: Probability - Validity of tool: Previously reported validated tool - Pilot testing: Not reported - Response rate: Not reported | - Country: USA - Participants: Male 1295 (56%) Female 1007 (44%) - White American 930 (40%) - Hispanic/Latino: 414 (18%), White Middle Eastern: 138 (6%), Indian Asian or Pakistani Asian: 213 (9%), Black or African American: 164 (7%) Others e.g. American Indian/mixed race: 439 (19%) - Setting: University Campus - N sampled: Unknown - N participated: 2334 - N analyzed: 2334 | - 47% of participants believed that waterpipe smoking was less harmful than cigarettes. - Answering the question: “Waterpipe is harmful to health” - Yes 1811 (80.7%), No 433 (19.3%) - “WTS is socially acceptable amongst peers” Yes 1457 (65%), No 783 (35%) - “WTS is Cool?” Yes 783 (35%), No 1434 (65%) |
| **16.**  **Factors associated with perceptions of hookah addictiveness and harmfulness among young adults.**  **Noonan et al.**  **2013** | - Sampling frame: University Students - Sampling Method: Simple Random - Recruitment method: Email - Administration method: Internet | - Sample size calculation: No - Sampling type: Probability Sampling - Validity of tool: Self developed tool no validation - Pilot testing:Not reported - Response rate: 270 (84%) | - Country: USA - Participants: University Students - Setting: University campus - N sampled: 320 - N participated: 270 - N analyzed: 270 | - Overall, 58.9% of the sample perceived WTS to be less addictive than cigarette smoking and 31.5% of the sample perceived it to be less harmful than cigarette smoking. - Students who perceived WTS to be less harmful than cigarette smoking tended to be older and were less likely to smoke cigarettes      - Students who perceived WTS to be less addictive than cigarette smoking were more likely to have smoked waterpipe in the past three months (32% vs. 11%, p<.001). |
| **17.**  **Harm perception of nicotine products in college freshmen.**  **Smith et al.**  **2007** | - Sampling frame: University Students - Sampling Method: Convenient sampling - Recruitment method: Email - Administration method: Internet | - Sample size calculation: No - Sampling type: Non-probability - Validity of tool: Self Developed tool - Pilot testing: Not reported - Response rate: 421 (49.5%) | - Country: USA - Participants: University students Mean age 18.7 Male 52% Female 48% White 58.4% - Setting: University - N sampled:421 - N participated: 411 - N analyzed:411 | - The question “WTS is less harmful than cigarettes” was answered yes by 37% |
| **18.**  **Hookah smoking: behaviors and beliefs among young consumers in the United States.**  **Griffiths et al.**  **2014** | - Sampling frame: University Students - Sampling Method: Purposive - Recruitment method: In person - Administration method: In person - interviewer administered | - Sample size calculation: Not reported - Sampling type: Non-Probability - Validity of tool: Self developed tool no validation - Pilot testing: Not reported - Response rate: 100% | - Country: USA - Participants:   University students  10 males and 10 females  aged between 18-24.   - Setting: University Campus - N sampled:20 - N participated: 20 - N analyzed:20 | - WTS carries a negligible health risk, that is much lower than smoking cigarettes. - Furthermore, WTS users did not believe their exposure to the addictive agents in tobacco was sufficient to create a significant addiction risks |
| **19.**  **Prevalence and correlates of waterpipe tobacco smoking by college students in North Carolina.**  **Sutfin et al.**  **2011** | - Sampling frame:   University students in North Carolina   - Sampling Method: Stratified random sampling - Recruitment method: Email - Administration method: Internet | - Sample size calculation: Not done - Sampling type: Non-probability - Validity of tool: Self developed tool adapted from Maziak and colleagues (2005) and Ward and colleagues (2007), - Pilot testing: None - Response rate: 26.9% | - Country: USA - Participants:   63% Females. Majority were white (80%). The sample had an even spread of the different student years (i.e. Freshman, Sophomores, Juniors and Senior)   - Setting: University Campus - N sampled: 3600 - N participated: 3370 - N analyzed:3370 | - 1174 (31%) believed that WTS was less harmful than cigarette smoking. 1881 (30%) believed that WTS is as harmful as cigarette smoking and 659 (17%) believed that it is more harmful. |
| **20. Assessing and Predicting Susceptibility to**  **Waterpipe Tobacco Use Among College Students**  **Lipkus et al.**  **2015** | - Sampling frame: Colleges in Virginia and North Carolina - Sampling Method: Convenience Sampling - Recruitment method: Email - Administration method: Internet | - Sample size calculation: No - Sampling type: Non-probability sampling - Validity of tool:   Self-developed, non-validated tool, but based on four items adapted from Pierce and colleagues.   - Pilot testing:   Not Reported   - Response rate:   35.6% from first screening survey.  64.2% from  Second  Screening  survey. | - Country: USA - Participants: 48.2% male. 81.8% white. 93.4% non-Hispanic. - Setting: 2012 - 2013 - N sampled: Not reported - N participated: 2375 - N analyzed: 964 | - In 2012, 14.9% believe they will smoke waterpipe soon, and 24.2% stated that they would smoke waterpipe if their friend offered it to them. - In 2013, 16.2% said they would smoke waterpipe soon, and 25.3% said if their friend asked them to smoke waterpipe they would. |
| **21.**  **A Theoretical Examination of Waterpipe Smoking in College Students.**  **Noonan et al.**  **2014** | - Sampling frame:   Student email  registry at  American college   - Sampling Method:   Simple Random  sampling   - Recruitment method: Email - Administration method: Internet | - Sample size calculation: No - Sampling type: Probability Sampling - Validity of tool: Previously used, validated tool - Pilot testing: Behavioral belief and normative belief scales were developed from pilot work. - Response rate: 53% | - Country: USA - Participants: The mean age of the sample was 19.9 (SD 1.3). Majority of the sample was Caucasian (76%), and more females (57%) completed the survey than males. 50% reported ever having smoked tobacco using a waterpipe - Setting: University college. Date unknown. - N sampled: 1000 - N participated: 261 - N analyzed: 223 initially, and then 120 completed the follow-up questionnaire. | - Having a positive perception of WTS increases the likelihood of its initiation over time. |
| **22.**  **Effectiveness of health warnings for waterpipe tobacco smoking among college students.**  **Islam et al.**  **2016** | - Sampling frame: Students at University of South Carolina - Sampling Method: Convenient - Recruitment method: IN person - Administration method: In person, self-administered | - Sample size calculation: No      - Sampling type: Non-probability      - Validity of tool: Self-developed tool, non-validated. Based on standard university-based waterpipe users      - Pilot testing: Not reported - Response rate: 69.6% | - Population: Mean age - was 21.9 years (SD. - 4.03). 50.4% of the cohort were male. - Most participants identified themselves as non-Hispanic white (68.1 %) and undergraduates (80.4 %) - Country: USA - Participants: 367 - Setting: University of South Carolina - N sampled: 525 - N participated: 367 - N analyzed: 367 | - 70.6 % believed cigarettes were more harmful than WTS, and 85.0 % believed cigarettes were more addictive than WTS. - 54.3 % believed cigarettes contained more nicotine than WTS. - 74.4 % believed that switching from cigarettes to WTS would reduce the health risks associated with using tobacco products. - A higher proportion of ever waterpipe users indicated that the warnings were very much motivational to quit smoking. - Best location for noticing a health warning label among ever users were the mouthpiece (41.2 %) followed by the stem (36.6 %). - The most effective locations of health warnings among current users were the base (32.4 %), followed by the mouthpiece (31.4 %) and the stem (30.5 %). |
| **23.**  **Hookah and Alcohol Use among Young Adult Hookah Smokers: A Mixed Methods Study.**  **Soule et al.**  **2015** | - Sampling frame: College and Non-College students at Virginia Commonwealth University - Sampling Method: Convenient Sampling - Recruitment method: Advertisements (mail) - Administration method: Internet Survey and in person, group discussion | - Sample size calculation: No      - Sampling type: Non-probability sampling      - Validity of tool: Self developed tool, no validation reported - Pilot testing: Not Reported - Response rate: Not Reported | - Country: USA - Participants: - Mean age of 19.2 (SD=1.4). Most of the participants (77.5%) were under the age of 21. The majority of the participants were female (55.0%), white (72.5%) and non-Hispanic (70.0%) - Setting: Virginia Commonwealth University - N sampled: N/A - N participated: N/A - N analyzed: 40 | - Prefer to smoke waterpipe while also drinking alcohol. |
| **24.**  **Perceived harm, addictiveness, and social acceptability of tobacco products and marijuana among young adults: marijuana, hookah, and electronic cigarettes win.**  **Berg et al.**  **2015** | - Sampling frame: Email addresses of all students two southeastern colleges - Sampling Method: Random sampling - Recruitment method: Email - Administration method: Internet | - Sample size calculation: No - Sampling type: Probability Sampling   Validity of tool: Self developed tool, no validation reported. Questionnaire adapted from other surveys e.g. National Youth Tobacco Survey   - Pilot testing: Not reported - Response rate: 20.0% | - Country: USA - Participants:   Average age 21.02 (SD =  2.02), 71.6% (n = 1,407)  females, and 40.0% (n =  787) Black.   - Setting: Spring 2013 - N sampled: 10,000 - N participated: 2,002 - N analyzed: 2,002 | - Products perceived to be least harmful to health were marijuana (4.14 ± 2.14), electronic cigarettes (4.26 ± 1.95), and waterpipe (4.56 ± 1.78) - Products perceived to be the most harmful were cigarettes (6.47±1.00), cigar products (6.19±1.19), and smokeless tobacco (6.07±1.30). - The products perceived to be the least addictive were waterpipe (3.66±2.12), electronic cigarettes (4.29±2.08), and marijuana (4.60±2.24); those perceived to be the most addictive were cigarettes (6.42±1.27), smokeless tobacco (5.63±1.72), and cigar products (5.63±1.72). - Products perceived to be the most socially acceptable were waterpipe (5.39±1.88), marijuana (5.13±2.06), and cigarettes (4.51±2.02); those perceived to be the least were smokeless tobacco (3.60±2.05), electronic cigarettes (4.12±2.03), and cigars (4.43±1.97). - Predictors of more favorable perceptions of WTS included more friends who smoke cigarettes (p = .05), more friends who use the waterpipe (p < .001), recent cigarette smoking (p = .009), and recent cigar smoking (p = .04 |
| **25.**  **Pilot Study of Waterpipe Tobacco Smoking Among US Muslim College Students.**  **Arfken et al.**  **2014** | - Sampling frame:   Respondent  driven sampling,  initiating with  Muslim student  organizations   - Sampling Method: Snowball sampling      - Recruitment method:   Unknown   - Administration method: Internet | - Sample size calculation: No - Sampling type: Non-probability - Validity of tool:   Self-developed  tool, no  validation  reported.  Questions  regarding  motivation for  waterpipe were  adapted from  alcohol use  motivations  questions in the  College Alcohol  Survey.  Questions regarding religiousness taken from US National Survey of Drug Use and Health (NSDUH) and one from a study on American Muslim physicians   - Pilot testing: Not reported - Response rate: Snowball until 156 students selected | - Country: USA - Participants:   67.9% female.  Predominately South  Asian (46.8 %) and Arab  (35.3 %).  Almost all (91 %) of the  sample graduated from  US high school with  about a third  (38.5 %) of the students  reporting that they  thought exclusively in  English. 82.1 % lived with  their parents and  received a scholarship  (60.3 %).   - Setting: N/A - N sampled: N/A - N participated: N/A - N analyzed: 156 | - 54.8 % of students who had ever smoked waterpipe did not rate any motivation as very important. Among the remaining students, top-rated items were to have a good time with my friends (24.7 %) and safer than cigarettes (20.5 %). - Part of my culture was rated as very important by only 4.2 %; this rating did not differ by ethnicity or nativity of the participant. - Lifetime waterpipe smoking was also strongly associated with perception that ‘‘most or all’’ of undergraduate students engage in waterpipe smoking (OR = 3.60). Ethnicity and nativity and ethnicity were not associated with waterpipe smoking. - Only 26.1 % of Muslim students reported waterpipe smoking was prohibited in Islam. - Believing that waterpipe smoking was prohibited in Islam was not a protective factor against lifetime waterpipe use (OR 0.68, 95 % CI 0.33–1.41), |
| **26.**  **Social influences on use of cigarettes, e-cigarettes, and hookah by college students.**  **Noland et al.**  **2016** | - Sampling frame:   Students on the  University  register in the South  Eastern USA   - Sampling Method:   Random Sampling     - Recruitment method:   Email   - Administration method: Internet | - Sample size calculation: No - Sampling type: Probability Sampling - Validity of tool:   Self-developed  tool, no  validation  reported.  For perceptions  of social norms,  authors used a  previously  validated tool   - Pilot testing: Not reported - Response rate: 13.6% | - Country: USA - Participants: 28.4% male. 80.2% white. 81.2% undergraduate. 52.4% live on-campus - Setting: April 2013 - N sampled: 4050 - N participated: 551 - N analyzed: 511 | - Scores for tobacco products that are considered “social norms” were highest for waterpipe, followed by e-cigarettes. - Cigarettes had the lowest social norms scores of the 3 products, and this was consistent for users and nonusers. - Cigarettes were perceived to be the most widely used among peers, followed closely by waterpipe. The perception of e-cigarette use among peers was about half as much as the other 2 products. - In the bivariate analysis, the waterpipe social norms score was associated with residence type, tobacco use, number of smokers in life, and reported exposure to second hand smoke. - Finally, perception of peer waterpipe use was associated with sex, race/ethnicity, and exposure to second hand smoke in the bivariate analysis. |
| **27.**  **The influence of drinking motives on hookah use frequency among young multi-substance users.**  **Foster et al.**  **2016** | - Sampling frame: Classrooms at a public University in Southern USA. Also, students receiving flyers on campus - Sampling Method: Convenient Sampling      - Recruitment method: In person - Administration method: Internet | - Sample size calculation: No - Sampling type: Non-probability - Validity of tool: Previously reported, Validated Tools - Pilot testing: Not Reported - Response rate: Not reported | - Country: USA - Participants:   134 (75.4%) female.  52.67 % Caucasian (of  these, 65.22 % identified  as non-Hispanic), 9.16 %  African American,  1.53 % Native American, 9.92% Asian, 9.92 % mixed, and 16.79% other.  Setting: College,   - N sampled: N/A - N participated: N/A - N analyzed: 134 | - Most participants believed that smoking waterpipe was somewhat harmful to health (70.70 %); however, only 8.30 % felt that waterpipe use was worse for health relative to alcohol or cigarettes |
| **28.**  **Future physicians and tobacco: an online survey of the habits, beliefs and knowledge base of medical students at a Canadian University.**  **Vanderhoek et al.**  **2013** | - Sampling frame:   Undergraduate Doctor of Medicine Students at the University of Alberta   - Sampling Method: Convenience - Recruitment method: Internet - Administration method: Email | - Sample size calculation: No - Sampling type: Non-probability - Validity of tool:   Self-developed,  non-validated  tool (based on  previously used  questionnaires  about tobacco  and education –  as well as input  from a  consultant in  social research  methodology.     - Pilot testing: No - Response rate: 44.2% | - Country: Canada – Edmonton - Participants:   51% were females. Mean  age was 24.4 years (SD = 2.8). About 37% of respondents had just completed their first year of medical school, while 14% of respondents had just graduated from their fourth year   - Setting: University - N sampled: 681 - N participated: 669 - N analyzed: 301 | - A significant minority of all respondents believed that smoking tobacco in a waterpipe was less harmful than smoking tobacco in the form of a cigarette. - 10% of those who had smoked a waterpipe indicated that they did not think that smoking tobacco in a waterpipe was addictive, a belief not shared by nonsmokers. |
| **29.**  **Predictors of Persistent Waterpipe Smoking Among University Students in The United States.**  **Abughosh et al.**  **2011** | - Sampling frame: Members University of Houston Student body - Sampling Method: Convenience - Recruitment method: Internet - Administration method: Internet | - Sample size calculation: No - Sampling type: Non-probability - Validity of tool:   Self-developed tool, non-validated, but based on previously used tools.   - Pilot testing: Not reported - Response rate: Not Reported | - Country: USA - Houston - Participants:   Half of the sample were  male (50.27%). 45% were  of white non Middle-Eastern ethnicity. Other ethnicities included Hispanic or Latino (18.24%), white Middle Eastern descent (8.37%), and Indian Asian or Pakistani Asian (8.19%), and others (19.38%) including Black/ biracial/American Indian/Other Asian.  Majority of the sample  (62.01%) were 22 years  old or older, and had  Middle Eastern  friends (75.68%).   - Setting: College, February 2011 - N sampled: N/A - N participated: N/A - N analyzed: 2204 | - 76% of the sample believed WTS was harmful to health but 57% believed it was less harmful than cigarettes. - Most of the sample thought there is no or low chance to get addicted when using a WTS socially (67%), and the majority (83%) viewed WTS as socially acceptable among peers. - Those who felt their peers look cool or very cool while smoking waterpipe were more likely to use waterpipe frequently (OR= 2.66). - Those who believed that waterpipe smoking was harmful to one's health were less likely to use waterpipe to smoke tobacco on a weekly basis (OR=0.30). |
| **30.**  **Waterpipe smoking among students in one US university: predictors of an intention to quit.**  **Abughosh et al.**  **2012** | - Sampling frame: Email registry of all undergraduate and graduate students on the University of Houston - Sampling Method: Convenience - Recruitment method: Email - Administration method: Internet | - Sample size calculation: No - Sampling type: Non-probability - Validity of tool:   Previously validated tool   - Pilot testing: Yes - Response rate: Not reported | - Country: United States - Participants:   42.0% of the  participants were  male and 54.1% were  younger than 22  years. 35.6% of the  Participants identified  themselves as white,  9.1% were Indian  Asian, 20.4% were  Hispanic, and 9.3%  were others  (including  black/biracial/America  Indian/other Asian).   - Setting: University of Houston, February 2011 - N sampled: ~38,000 - N participated: - N analyzed: 2,204 | - The majority of the sample (n = 227; 83.2%) reported no intention to quit waterpipe smoking with only 5.1% (n = 14) reporting an intention to quit in the next year. - 67.5% (n = 185) of the sample believed waterpipe smoking was harmful to health, but 60.9% (n = 168) believed it was less harmful than cigarettes. - The majority of the sample (n = 260; 94.2%) viewed waterpipe smoking as socially acceptable and cool (n = 199; 73.7%) and thought there is no or low chance to get addicted when using a waterpipe socially (n = 209; 76.3%) or alone (n=176; 64.0%). - Participants who believed that waterpipe smoking was harmful to one’s health were more likely to have a desire to quit (OR = 2.38, CI [1.05, 5.36]. - Participants who indicated they smoked the waterpipe for more than 1 hour each time they smoked were less likely to have an intention to quit compared with those who smoked for a lesser period of time (less than 30 minutes) (OR = 0.29, CI [0.12, 0.73]). - Participants with Indian or Pakistani descent (n = 25) were more likely to have an intention to quit compared with white non-Middle Eastern descent participants (OR = 4.74, CI [1.61, 13.93]), but this was based on a very small sample size. |
| **31.**  **Hookah smoking behavior initiation in the context of Millennials.**  **Castaneda et al.**  **2016** | - Sampling frame: Young adult waterpipe smokers - Sampling Method: Convenience sampling - Recruitment method: Mail - Administration method: In person, group discussion | - Sample size calculation: No - Sampling type: Non-probability sampling - Validity of tool: Non-validated, but based on previous tools (Based on past work using outcome expectant theory to cigarettes) - Pilot testing: Not Reported - Response rate: Not reported | - Country: United States - Participants: Mean   age of 19 years (SD 1.4). 55% females. Predominantly white (72%), followed by Asian (10%), and black (7.5%), 30% Hispanic, and 12.5% Middle Eastern.  Additionally,  occupation status was  fulltime students  (75.5%); with  Part-time workers  (15%), fulltime  workers (10%), and  unemployed  individuals (5%).  .   - Setting: Group discussion in 5 – 9 participants - N sampled: N/A - N participated: N/A - N analyzed: 40 | - Millennial waterpipe users had commonly held beliefs that waterpipe use was nonaddictive and viewed their recurrent use as a transient behavior that was a part of their college experience. - One participant noted he had discontinued use after his initial waterpipe smoking attempt until after he arrived to the college   environment. |

| **32.**  **Waterpipe smoking: prevalence and attitudes among medical students in London.**  **Jawad et al.**  **2013** | - Sampling frame:   1^st^, 2^nd^ and 5^th^ year  medical students at  Imperial College, London   - Sampling Method: Not Stated - Recruitment method: Not stated - Administration method:   In person, self-administered | - Sample size calculation: No - Sampling type: Unknown - Validity of tool: Previously reported validated tool - Pilot testing: Not reported - Response rate: Not reported | - Country: United Kingdom   Participants: Mean age of the sample was 20.4 ± 2.0 years and 54.6% were female. Most respondents were from a White or South Asian (respectively 38.0% and 36.6%) ethnic background, and 10.6% were Chinese; 42.0% reported a total household income of between approximately US$60 000 and US$160 000   - Setting: Imperial College London. 2011 - N sampled: N/A - N participated: N/A - N analyzed: 489 | - 60.0% revealed no intention to quit. - Furthermore, 45.2% of all respondents stated that WTS was less harmful than cigarettes or not harmful at all, and 32.7% intended to smoke it in the future, although only 18.8% would encourage their peers to smoke it |
| --- | --- | --- | --- | --- |
| **33.**  **Waterpipe tobacco smoking on a U.S. College campus: prevalence and correlates.**  **Eissenberg et al.**  **2008** | - Sampling frame:   Students enrolled in  Virginia Commonwealth  University Introduction to  Psychology courses   - Sampling Method: Convenient - Recruitment method: Not Reported - Administration method: Internet | - Sample size calculation: No - Sampling type: Non-probability - Validity of tool: Previously used, validated tool - Pilot testing: N/A - Response rate: 62.3% | - Country: United States - Participants: 65% were women, 72% were 18 or 19 years old, 57% were White, and 20% had smoked a waterpipe in the past month. - Setting: Virginia Commonwealth University, March 2006 - N sampled: N/A - N participated: 744 - N analyzed: 744 | - Users of waterpipe had increased odds of future use compared to never-users if they believed that it made them look cool (OR 2.47) or that its use was socially acceptable amongst peers (OR 3.71). - Students’ believe WTS causes less harm than cigarette smoking. (OR = 0.31). |
| **34.**  **Waterpipe Tobacco Use in the United Kingdom: A Cross-Sectional Study among University Students and Stop Smoking Practitioners.**  **Jawad et al.**  **2016** | - Sampling frame: Enrolled undergraduate and postgraduate students across 6 UK universities - Sampling Method: Convenience Sample - Recruitment method: Internet as some universities, email at others to the students   Email to the stop smoking practitioners   - Administration method:   Internet | - Sample size calculation: No - Sampling type: Non-probability sampling - Validity of tool: Self developed tool, no validation reported - Pilot testing: Not reported - Response rate: Not reported | - Country: United Kingdom - Participants:   Mean 23.4 years. 59.6% from UCL. 58.4%  female and 74.9% white. 59.9%  undergraduates   - Setting: University College London, Imperial College London, Kings College London, University of York, University of Cardiff, University of West of England. - N sampled: N/A - N participated: N/A - N analyzed: 2213 | - Regarding policy, a third reported ever violating the smoke free law since its implementation, a quarter ever noticed health warnings on waterpipe tobacco packaging or on waterpipe apparatuses, and one in ten reported ever being informed of the safety of waterpipe smoking from watepipe café staff or marketing material - Compared to less than monthly users, those using at least monthly were significantly more likely to, report difficulty in quitting waterpipes (0.8% vs. 15.5%, χ p<0.001), report feeling annoyed when people criticized waterpipe smoking habits or told them to quit waterpipe (9.5% vs. 32.2%, χ p<0.001), report feeling guilty about waterpipe smoking (9.2% vs. 19.2%, χ p<0.001), and report ever having tried to stop smoking waterpipe (4.3% vs. 12.4%, χ p<0.001). |
| **35.**  **Antecedents of university students' hookah smoking intention.**  **Martinasek et al.**  **2013** | - Sampling frame:   Students at a South Eastern USA university   - Sampling Method: Simple random sampling (for questionnaire)   Convenient sampling for focus groups.   - Recruitment method: Focus group by mail/flyers.   Questionnaire by internet   - Administration method:   Questionnaire by internet.  Focus group conucted in person, group discussion | - Sample size calculation: Yes - Sampling type: Questionnaire – probability sampling. - Interview – Non-probability sampling - Validity of tool: Self developed tool, validation reported - Pilot testing:Yes - Response rate: 24.6% | - Country: USA - Participants:   72.3% women . Most respondents 18- 19 years of age (71.8%) and White (68.3). Most commonly reported religion  was “other Christian” (30.3%).  .   - Setting: Spring break, 2011 - N sampled: 1500 - N participated: N/A - N analyzed: 369 | - Personal attitudes correlate more with future intention to smoke waterpipe than students’ perception of the subjective norm. (rho 0.792 vs rho 0.445 respectively, p < 0.01) - There is a negative correlation between attitude towards WTS and health effects. |

| **36.**  **A qualitative analysis among regular waterpipe tobacco smokers in London universities.**  **Jawad et al.**  **2013** | - Sampling frame:   University students in London   - Sampling Method: Snowball Sampling - Recruitment method: Telephone, Email and Social Media - Administration method: In person, group discussion | - Sample size calculation: No - Sampling type: Non-probability sampling - Validity of tool: Not reported - Pilot testing: Not Reported - Response rate: Not reported | - Country: UK - Participants:   Majority were male (75%), of South Asian (46.9%) or Arab (43.4%) ethnicity, and smoked the waterpipe less than weekly but at least monthly (43.4%);   - Setting: Interviews conducted in universities or meeting rooms. January – April 2012. - N sampled: N/A - N participated: N/A - N analyzed: 32 | - Positive attitudes such as a pleasant sensory experience, social acceptability, socializing and its use as a social lubricant were all factors that contributed to the use of waterpipe. - Participants cited non-specific neurological and respiratory health effects after using WTS. For those who smoked both cigarettes and waterpipe, many agreed that cigarettes are worse. - Many felt the evidence/arguments detailing the negative health effects of WTS were weak and there were no coherent health promotion campaigns to educate them on this. - Participants believe that quitting WTS is easy- although all waterpipe smokers who had attempted to quit in the past had failed to do so - Participants general believed WTS to be a ‘social addiction’ |
| --- | --- | --- | --- | --- |
| **37.**  **Attitudes and practices of hookah smokers in the San Francisco Bay Area.**  **Ahmed et al.**  **2011** | - Sampling Frame UC Berkley students - Sampling Method:   Convenience Sample   - Recruitment method:   Email and Mail   - Administration method: In person – whether interviewer administered or self-administered not reported | - Sample Size Calculation: Not reported - Sampling type: Non-probability - Validity of tool:   Self Developed Tool – no validation reported   - Pilot testing:   Not repoted   - Response rate: Not Reported | - Country: America - Participants:   Students at UC Berkeley. Average age 22.9 years (mean). 28% had completed their undergraduate college degree. 58% of the cohort were Asian in origin and 26% were white   - Setting: UC Berkeley - N sampled: Not Reported - N participated: Not Reported - N analyzed: 50 | - The majority of respondents considered WTS to be harmful to their health (88%), yet 52% had no intention of quitting - Students believed waterpipe smoking was equally or more harmful than cigarette smoking |
| **38.**  **Affecting perceptions of harm and addiction among college waterpipe tobacco smokers.**  **Lipkus et al.**  **2011** | - Sampling Frame:   University students at 6 college and university campuses in North Carolina (those who responded to an advertisement in these campuses)   - Sampling Method:   Convenience Sample   - Recruitment method:   Mail (advertisements)   - Administration method: Internet | - Sample Size Calculation: Not Reported - Sampling type:   Non-probability sampling   - Validity of tool:   Self-developed tool, non-validated   - Pilot testing:   Not reported   - Response rate: Not reported | - Country: America - Participants:   This study had 2 parts, with differing characteristics of the patients responding at the first stage and the second stage.  In general, mean age was 20.5, with ~ 28% women. Majority of the sample was Caucasian (~70%).   - Setting: North Carolina College - N sampled: Part 1: 177, Part 2: 153 - N participated: Part 1: 108, Part 2: 126 - N analyzed: Part 1: 108, Part 2: 126 | - Students who received information about harms and exposures of WTS reported greater perceived personal health risk and expressed more worry compared to those who did not. - Students are aware of the negative effects of WTS - Motives for WTS use included: Curiosity, Peer influence, smell, liking the way the waterpipe is crafted, and the convenience of waterpipe cafes nearby |
| **39.**  **Hubble bubble trouble: the need for education about and regulation of Hookah smoking.**  **Griffiths et al.**  **2011** | - Sampling Frame: Students at WTS café’, but also students at a large southeastern university. - Sampling Method:   Initially, convenience Sampling, then followed by snowball sampling   - Recruitment method:   Not clear – may be in person?   - Administration method:   In person, group discussion | - Sample Size Calculation: Not reported - Sampling type: Non-probability sampling - Validity of tool: Self developed tool, no validation reported - Pilot testing: Not reported - Response rate: Nit reported | - Country: America - Participants: 20 participants were recruited (10 Male). Average age was ~ 20m, and students were generally Juniors. Majority were causcasian. - Setting: Wherever participants felt comfortable (including college campuses and WTS café’s). - N sampled: Not reported - N participated: Not reported - N analyzed: 20 | - Reasons for WTS use include: Relaxation. Peer influence, curiosity, - University students who smoked waterpipe, respondents believed that the addictive effect of WTS is unlikely because of its occasional occurrence |
| **40.**  **Prevalence, risk factors, symptoms of addiction, and smoke intake: evidence from one British university.**  **Jackson et al.**  **2008** | - Sampling Frame:   Students at University of Birmingham, UK   - Sampling Method:   Convenience Sampling   - Recruitment method:   In person (but by just distributing the questionnaires in lectures)   - Administration method:   In person, unsure as to whether self or interviewer administrated. | - Sample Size Calculation:   Not reported   - Sampling type:   Non-probability sampling   - Validity of tool:   Self-developed tool, no validation reported   - Pilot testing:   Not reported   - Response rate: Not reported (but they say they wanted to study around 1000, and 937 completed the survey’s). | - Country: United Kingdom - Participants: Students at the University of Birmingham. Mean age 20.2 years. 37.9% had tried WTS. - Setting: Questionnaires were conducted in the University - N sampled: ~1000 - N participated: 937 - N analyzed: 937 | - Majority started WTS after being introduced by a friend or by family. - Of these, 17 (81.0%) intended to carry on smoking after the ban on smoking in enclosed public spaces was introduced (which was imminent at that time) and 15 (71.4%) smoked waterpipes at home. - All but one smoker felt WTS was socially acceptable. 19 (90.5%) thought WTS was bad for health, but of these, 13 (68.4%) thought waterpipes were less damaging than cigarettes. - Two (9.5%) regular waterpipe smokers had tried to stop smoking waterpipes but restarted. Seven (33.3%) had experienced cravings to smoke waterpipes. |

| **41.**  **College students' perceptions and knowledge of hookah use.**  **Creamer et al.**  **2016** | - Sampling frame:   University Emailing List   - Sampling Method:   Convenience Sampling   - Recruitment method:   Email   - Administration method:   Internet | | - Sample size calculation: None      - Sampling type:   Non-probability Sampling   - Validity of tool: - Pilot testing:   No   - Response rate:   5482 (40%) | - Country: America - Participants:   Adult students aged 18-29 years. 63.4% were female and 36.3% were non-hispanic white     - Setting: Colleges across Texas - N sampled: 13714 - N participated: 5482 - N analyzed: 5482 | - No difference between users and non-users in beliefs of hookah’s tobacco content - In multivariate logistic regression models, knowledge of hookah contained tobacco was associated with increased odds of current hookah use and harms perceptions regard hookah use were associated with decreased odds of current use. - Knowledge of hookah contents was **not** associated with current hookah user’s intensity of use - Increased perceptions regarding addictiveness were associated with higher intensity of use. |
| --- | --- | --- | --- | --- | --- |
| **42.**  **Urban college student self-report of hookah use with health care providers.**  **Jani et al.**  **2018** | - Sampling frame:   Students in the lunch queue   - Sampling Method:   Systematic Random Sampling   - Recruitment method:   In person   - Administration method:   In person (method of administration not discussed) | | - Sample size calculation:   Yes (power calculation conducted)   - Sampling type:   Probability Sampling   - Validity of tool:   Self-developed, non-validated tool (but based on prior literature)   - Pilot testing:   Not reported   - Response rate:   402 participants. (89%). | - Country:   America   - Participants:   Majority white participants (45.3%), with equal percentages between 18-20 (49.3%) and > 21 (50.7%).     - Setting:   College canteen   - N sampled: 454 - N participated: 402 - N analyzed: 362 | - Students who believed WTS was healthier than cigarettes had higher odds of WTS use (2x) than reference (belief that WTS and Cigarettes are equal risk). - Perceived socialibility of WTS vs. cigarettes didn’t not influence odds of use. But most reported that WTS is more acceptable and healthier than cigarettes.   . |
| **43.**  **University students' perceived risk of and intention to use waterpipe tobacco.**  **Rayens et al.**  **2017** | - Sampling frame:   Students on the Email registrar   - Sampling Method:   Convenience Sampling   - Recruitment method:   Email   - Administration method: Internet | | - Sample size calculation:   No   - Sampling type:   Non-probability Sampling   - Validity of tool:   Self developed tool – no validity reported   - Pilot testing:   No   - Response rate: 15.4% | - Country:   America   - Participants:   Majority of participants were female (72%) and White/Non-Hispanic (79%). Largest subgroup were freshman or sophomore (61%) and ¼ were graduate students   - Setting:   University of Kentucky   - N sampled: 5000 - N participated: 741 - N analyzed: 667 | - Amongst 218 users, the frequent reasons for using WTS were to ‘socialise’ (90%) and ‘liking the taste (47%)’. - 16.7% believed it was less addictive than cigarettes and 15.4% believed it was less harmful to others. - 5.2% stated cultural reasons and 5.0% said it was due to that they can use it where smoking is not allowed - Those who had established use of ‘Tobacco Products’ had significantly less risk of commencing WTS use - Having a belief it was socialible significantly decreased the odds of believing WTS could be harmful - A greater intention ot commence WTS was seen in students who perceived WTS to be social acceptable, less risky and former tobacco users (inc. cigarettes) |
| **44.**  **Descriptive and injunctive norms of waterpipe smoking among college students.**  **Leavens et al.**  **2018** | | - Sampling frame: University students - Sampling Method: Convenience Sampling - Recruitment method: Internet - Administration method: Internet | - Sample size calculation: Not reported - Sampling type: Non-probability - Validity of tool: Self developed tool, no validation reported - Pilot testing: Not reported - Response rate: 100% | - Country: USA - Participants: University Students - Mean age 19.64.   Male N=300 (33.6%) Female N=594 (66.4%)   - Setting: University campus - N sampled: 975 - N participated: 975 - N analyzed:894 | - Close to half (47.6%) of never users reported that none of their five closest friends would approve of their WTS compared with only 19.9% of ever users believing that none of their closest friends would approve of their smoking. 43.4% of ever users reported that all five of their five closest friends would approve of their smoking WP, while only 16.4% of   never users believed that five of their five closest friends would approve. |
| **45.**  **Hookah Smoking: Assessing College Students' Behaviors, Attitudes, and Knowledge.**  **Krenik-Matejcek et al.**  **2017** | | - Sampling frame: University Students - Sampling Method: Convenience - Recruitment method: In Person - Administration method: In person self administered | - Sample size calculation: No reported - Sampling type: Non-probability - Validity of tool: Self developed tool. Not validated - Pilot testing: Yes - Response rate: 98% | - Country: USA - Participants: University Students - Setting: University Campus - N sampled: 204 - N participated: 200 - N analyzed:200 | - Over half of participants (54%) stated hookah smoking is socially acceptable, with 21% reporting positive social benefits. Almost half of participants (43%) believed hookah smoking has relaxation benefits. Users perceive greater positive social benefits from hookah smoking (25%) than non-users (11%). As expected, more users believe hookah smoking is socially acceptable (68%) than non-users (22%). However, the majority of both users (67%) and non-users (69%) stated that hookah use is increasing in popularity. - Hookah smoke is filtered through water so some of the harmful ingredients get filtered out – correct answer FALSE – Users correct 59 (43.4%) Non-users correct19 (29.7%) - Smoking hookah is not as addictive as smoking cigarettes - FALSE – Users correct 49 (36%) Non-users correct 26 (40.6%) - Hookah contains higher levels of tar and carbon monoxide than cigarettes - TRUE – Users correct 41 (30.1%) Non-users correct 10 (15.6%) - Hookah smoking delivers nicotine – TRUE - Users correct 70 (51.5%) Non-users correct 33 (51.6%) - Smoking hookah began in the early 1900’s FALSE – Users correct 32 (23.5%) Non-users correct 6(9.4%) - Second-hand smoke from hookahs is not considered a health risk - FALSE – Users correct 82 (60.3%) Non-users correct 31 (48.4%) - Hookah can cause clogged arteries and heart disease - TRUE – Users correct 73 (53.7%) Non-users correct 28 (43.8%) - Hookah tobacco and smoke are known to cause oral cancers - TRUE – Users correct 90 (66.2%) Non-users correct 32(50%) - Sweetened and flavoured non-tobacco products sold for use in hookahs are less harmful than non-sweetened - FALSE – Users correct 68 (50%) Non-users correct 33 (51.6%) - A single hookah-smoking session typically involves inhaling less smoke volume than smoking several cigarettes - FALSE – Users correct 75 (55.1%) Non-users correct 26 (40.6%) |
