# Supplementary material for: Knowledge, attitudes, and perceptions towards waterpipe tobacco smoking amongst college or university students: a systematic review
Source: BMC Public Health. 2019 Apr 27;19:439. doi: 10.1186/s12889-019-6680-x (PMC6487066; doi:10.1186/s12889-019-6680-x)
Supplement: Supplementary file 4 — Global South Studies. Characteristics of all included Global South Studies. (DOCX 111 kb) [file 12889_2019_6680_MOESM4_ESM.docx]

| **1.**  **Comparison of cigarette and water pipe smoking among female university students in Egypt.**  **Labib et al.**  **2007** | - Sampling frame: WTS cafe's near two universities in Cairo - Sampling Method: Purposive sampling - Recruitment method: In person - Administration method: In person | - Sample size calculation: No power calculation      - Sampling type: Non-probability sampling - Validity of tool: Self developed tool, no validation reported. - Pilot testing: Not reported. - Response rate: 100% | - Country: Egypt (Cairo) - Participants:   Female University students.  Mean age: 21 (for medical  students), 20 (non-medical students).     - Setting: Cafe - N sampled: 196 - N participated: 196 - N analyzed: 196 | - Two universities sampled; but there was no significant difference in knowledge between the two (13% vs. 14% had good knowledge; whilst 32% vs. 42% had poor knowledge). - Common reasons for smoking WTS over cigarettes are: (1) perception that WTS is fashionable. (2) Belief it's less harmful than cigarettes. (3) Desire to be with friends in cafe. - Pleasure, curiosity, following friends are common reasons for WTS amongst these female university students. Others are a desire to look attractive and also feel free to make own life decisions. - 50% want to quit WTS - BUT this statistic is just to quit their tobacco habit, so could include cigarettes. - Curiosity was a significant factor for initiation (OR = 2.8, 95%, CI = 1.3 - 6.2, P < 0.01). |
| --- | --- | --- | --- | --- |
| **2.**  **Prevalence, social acceptance, and awareness of waterpipe smoking among dental university students: a cross sectional survey conducted in Jordan.**  **Obeidat et al.**  **2014** | - Sampling frame:   Students from dental related fields that include Dental (D), Dental Hygiene (DH) and Dental Technology (DT) students at Jordan University of Dental Sciences   - Sampling Method:   Convenience   - Recruitment method: In person - Administration method: In person, self administered | - Sample size calculation: Not Reported - Sampling type: Non-probability sampling - Validity of tool: Self-developed tool, validity not reported - Pilot testing: Yes - Response rate: 70.1% | - Country: Jordan   Participants:  7.6% were males,  69.8% were from the  dental school, and  30.2% were from  dental hygiene and  dental technology  departments. 30.2%  were 20 years of age  and 25.6% were 21  years of age. 37.5%  were in 4th year of  higher, and 35.1%  were in third year.   - Setting: Jordan University of Science and Technology - N sampled: 780 - N participated: 547 - N analyzed: 547 | - 61.7% of males and 45% of females in the study included ‘encouragement by friends’ as a reason for smoking shisha. The same proportion of men but 59% of women sought to smoke shisha for ‘pleasure’. - Around a third of students believed WTS to be less destructive to oral health than cigarette smoking. 82% of students identified WTS as causing carcinoma, 89% gum inflammation, 83.4% teeth staining. - Less were aware of WTS associated dental implant failure (52.5%), decay (64.1%) and halitosis (70.3%). |
| **3.**  **Role of members of university students' unions in tobacco prevention.**  **Allam et al.**  **2007** | - Sampling frame:   Members of Ain Shams  University Students’  Unions   - Sampling Method:   Simple Random Sampling (from different faculties in Ain Shams University)   - Recruitment method: In person - Administration method: In person, interviewer administered | - Sample size calculation: No - Sampling type: Probability Sampling - Validity of tool: Self developed tool, no validation reported - Pilot testing: Yes - Response rate: Not reported | - Country: Egypt - Participants: Members of Ain Shams University Students Union from March 1, 2005 to April 30, 2005. All male (all 108) - Setting: University - N sampled: N/A - N participated: 108 - N analyzed: 108 | - Regarding knowledge about Shisha smoking hazards, the majority of the students (84.3%) knew that Shisha smoking is hazardous. However, only 60 (55.6%) of the students knew that Shisha smoking is more hazardous than cigarette smoking. |
| **4.**  **Shisha smoking and associated factors among medical students in Malaysia.**  **Al-Naggar et al.**  **2012** | - Sampling frame:   Medical students from International Medical School, Management and Science University, Malaysia.   - Sampling Method: Simple random sampling - Recruitment method: In person - Administration method: In person – but whether interviewer or self-administered unknown. | - Sample size calculation: No - Sampling type: Probability sampling - Validity of tool: Self developed tool, no validation reported - Pilot testing: No reported - Response rate: Not reported | - Country: Malaysia - Participants:   Mean age was 22.5±2.5 years old, with maximum age of 32 years old and minimum age of 18 years old. The majority of the study participants were female, Malay, single, from year one and from urban areas (67%, 54%, 97%, 43.7%, 73%; respectively).  Setting: International Medical School, Management and Science University, Malaysia. December 2011 - March 2012.   - N sampled: N/A - N participated: 300 - N analyzed: 300 | - Some study participants were found to believe that shisha does not contains nicotine, does not contain carbon monoxide, does not lead to lung cancer, does not lead to dental problems, does not lead to cardiovascular diseases (25%, 20.7%, 22.3%, 29%, 26.7%; respectively). - 19% of the study participants did not believe that shisha is harmful to health and that 97% of the study participants believed that water in shisha “filters” toxins - The study also revealed that family problems, problems with friends, financial problems were significantly associated with shisha smoking status |
| **5.**  **Smoking, awareness of smoking-associated health risks, and knowledge of national tobacco legislation in Gaza, Palestine.**  **Shomar et al.**  **2014** | - Sampling frame:   Students from 7 universities in Gaza, Palestine   - Sampling Method: Convenient sampling - Recruitment method: Unknown - Administration method: In person, self-administered | - Sample size calculation: No - Sampling type: Probability Sampling - Validity of tool: Previously used, validated tool - Pilot testing: Not Reported - Response rate: 96% | - Country: Gaza, Palestine - Participants:   Students from 7  universities in Gaza,  Palestine  There were  Equal numbers of  males and females  (Mean age 22 ± 2.6  years).  Participants  represented many  disciplines,  categorized broadly  into literary faculties  (n = 650, 61.3% of  sample) and scientific  faculties (n = 408,  38.5%)   - Setting: May 2013. University - N sampled: 1104 - N participated: 1060 - N analyzed: g | - 6.8% believed that WTS is also dangerous to health. - The most reported health problems were cancer, respiratory diseases, and heart and vascular diseases |
| **6.**  **The frequency of shisha (waterpipe) smoking in students of different age groups.**  **Basir et al.**  **2014** | Sampling frame: Undergraduate and postgraduate students who were selected from institutions in Karachi   - Sampling Method: Unknown - Recruitment method: Unknown - Administration method: Unknown | - Sample size calculation: No - Sampling type: Unknwon - Validity of tool:   Self-developed tool. No validation reported   - Pilot testing: No reported - Response rate: Unknown | - Country: Pakistan - Participants:   The average age was 25±1.215 years  and frequency of shisha smoking was 48% (n=96)   - Setting: 2012 to February 2013. Undergraduate and postgraduate students - N sampled: Unknown - N participated: Unknown - N analyzed: 200 | Reason cited for shisha smoking:   - Social Activity - 50% - Addictive habit - 20.7% - Status Symbol – 12.2% - More tolerable then cigarettes – 15.9%   31.4% of the cohort knew that  shisha was injurious to health |
| **7.**  **Water pipe tobacco smoking among university students in Jordan.**  **Azab et al.**  **2010** | - Sampling frame:   Students of four universities in Jordan—Jordan University of Science and Technology (JUST), Yarmouk University, Irbid National University, and Jerash Private University.   - Sampling Method:   Two stage sampling method: 1st stage Cluster random sampling. Second stage Systematic random sampling.   - Recruitment method: Not reported - Administration method:Not reported | - Sample size calculation: No - Sampling type: Probability Sampling - Validity of tool: Self developed tool, no validation reported, but based on previously used tools and expert opinion.   “questionnaire was developed from standard instruments used previously to assess water pipe tobacco smoking (Eissenberg, Ward, Smith-Simone, & Maziak, 2008; Maziak, Ward, Afifi Soweid, & Eissenberg, 2005”   - Pilot testing: Not reported - Response rate: 74 – 78% across the 4 universities | - Country: Jordan - Participants:   51.8% (n = 282) were male. 92.8% (n = 504) were Jordanian, 92.0% (n = 494) were single, and 85.7% (n = 460) lived with family.  Mean age was 21.7 years (SD = 2.9), and mean monthly income was 652 JD (ca. 925 US dollars).  Students roughly equally represented each of the four study sites.  Most participants indicated that they were majoring in arts (36.3%), general sciences (30.4%), or medicine.   - Setting: 4 Universities across Jordan. March – July 2008 - N sampled: 735 - N participated: 552 - N analyzed: 548 | - The majority of the sample (62.2%) believed that WTS is more harmful than cigarette smoking, whereas only 9.8% believed that cigarettes are more harmful. The remaining 28.0% felt that harm was about the same. - With regard to addiction, the majority felt that cigarettes are more addictive than water pipe (54.6%), with only 13.2% sensing that water pipe was more addictive and 32.2% believing that addic­tive potential is about the same. - Although those believing that cigarettes were more harmful than water pipe were more commonly water pipe tobacco smok­ers, this relationship was only statistically significant for the outcome variable of water pipe use at least monthly (p < .001) and not for the outcome of ever use (p = .09). - Those believing that cigarettes were more addictive than water pipe were more commonly water pipe tobacco smokers (p < .001 for both outcomes—use at least monthly and ever use. |
| **8.**  **Water pipe (shisha) smoking and associated factors among Malaysian university students.**  **Al-Naggar et al.**    **2011** | - Sampling frame:   University students at Management and Science University, Shah Alam, Selangor, Malaysia   - Sampling Method: Convenient - Recruitment method: In person - Administration method: In person, self-administered | - Sample size calculation: No - Sampling type: Non-probability sampling - Validity of tool: Self developed tool, no validity reported, but based on a literature review - Pilot testing: Not reported - Response rate: Not reported | - Country: Malaysia - Participants:   majority of them were male, in the age group 20-22 years old, single, Malay, with income less than 2000 Ringgit Malaysia, from urban areas. (61.5%, 33.5%, 94.5%, 66%, 41.5, 76.5%   - Setting:   2010/ 2011 in Management and Science University (MSU), Shah Alam, Selangor, Malaysia   - N sampled: N/A - N participated: N/A - N analyzed: 200 | - 48.5% believe WTS to be less harmful than cigarettes. - 58.5% stated the water within the shisha apparatus filters the toxins. - 59.5% stated there being no tar in WTS - 77.5% stated WTS can cause cardiovascular and respiratory disease. - 66% of the sample considered the act of smoking shisha to be less addictive than cigarette smoking |
| **9.**  **Waterpipe (narghile) smoking among medical and non-medical university students in Turkey.**  **Poyrazoglu et al.**  **2010** | - Sampling frame:   Students of the first  three grades of the  medical faculty and the  engineering faculty  of Erciyes University   - Sampling Method: Convenience - Recruitment method: In person - Administration method: - In person, self-administered under the supervision of the investigators | - Sample size calculation: No - Sampling type: Non-probability testing - Validity of tool: Self developed tool, no validity reported - Pilot testing: Not reported - Response rate: 71.7% | - Country: Turkey - Participants: Medical and Engineering students at Erciyes University between 2008 - 2009 - Setting: Average age 20.3 years, 57.8% male, 55.3% from medical department, 45% Grade I, 26.4% grade 2 and 28.7% grade 3. 68.4% live in urban areas. 58% moderate economic status, and 37.8% good (4.2% poor) - N sampled: 908 - N participated: 651 - N analyzed: 645 | - The majority of the waterpipe users (91.0%) do not believe they are ‘hooked’ or dependent on the waterpipe - Most of the students thought that WTS is less addictive than cigarette smoking. Of the waterpipe users 61% stated that its health damage for the smokers and 41% stated that its harmful effect for other people are greater than cigarettes. These percentages were low among non-smokers of waterpipe (P < 0.05). - Approximately half of the waterpipe nonusers were undecided about the health damage for the smokers, and one-third of them were undecided about the harmful effect for other people. |
| **10. Waterpipe dependence in university**  **students and effect of normative beliefs:**  **a cross-sectional study.**  **Salameh et al.**  **2013** | - Sampling frame:   Lebanese students in the public and private universities.   - Sampling Method: Convenience Sampling - Recruitment method: In person - Administration method: In person, self administered | - Sample size calculation: No - Sampling type: Non-probability sampling - Validity of tool: Previously reported, validated tool - Pilot testing: Yes - Response rate: Not reported | - Country: Jordan - Participants: 58.5% male. 95.8% single. 42.1% aged between 20 – 21. 51.8% private universities, 48.2% public university. - Setting: - N sampled: N/A - N participated: 3384 - N analyzed: 3384 | - Among WTS students, 35% declared having the intention to stop smoking later, and 20% declared wanting to stop smoking   immediately.  Moreover, 28.7% ever tried to stop smoking but did not succeed. |
| **11.**  **Waterpipe tobacco use among Iranian university students: correlates and perceived reasons for use.**  **Sabahy et al.**  **2011** | - Sampling frame:   Students of two major universities in Kerman, Iran   - Sampling Method: Simple Random Sampling - Recruitment method: In person - Administration method:   In person, self-administered | - Sample size calculation: No - Sampling type: Probability Sampling   Validity of tool: Self developed, non-validated tool. Based on an extensive literature review and the comments and critiques of an expert panel   - Pilot testing: Not reported - Response rate: 91% | - Country: Iran - Participants:   Participants had a mean (±SD) age of 20.6 (±2.3); 517 (50.5%) were female. 8.0% married and 40.7% studying medicine  The lifetime prevalence of waterpipe smoking was 42.5%, and 18.7% of the sample were current waterpipe smokers   - Setting: Two universities in Kerman (the capital of the largest province of Iran). Date unknown. - N sampled: 1130 - N participated: 1024 - N analyzed: 1024 | - Most important reason for smoking shisha were: pleasure (55.8%), dealing with depression (22.1%), stress relief (9.0%), peer pressure (8.2%) and dealing with anger (4.9%). |
| **12.**  **Knowledge, attitudes and practice of university students regarding waterpipe smoking in Pakistan.**  **Jawaid et al.**  **2008** | - Sampling frame:   Students at 4 universities in Karachi   - Sampling Method: Simple random sampling - Recruitment method: Not reported - Administration method: Not reported | - Sample size calculation: Yes - Sampling type: Probability Sampling - Validity of tool: Self developed tool, non-validated (but based on other instruments) - Pilot testing: Not reported - Response rate: 92% | - Country: Pakistan - Participants:   59.6% males. Mean age - 21 years (S.D. 2.2 years); 59.3% (n = 267) of the participants were medical students, and the remainder (40.7%, n = 183) were students training for other professions   - Setting: 4 universities in Karachi, Pakistan. March 2006 – March 2007. - N sampled: 487 - N participated: 450 - N analyzed: 450 | - 61.4% (n=148) cited ‘curiosity’ as the main reason for initiating WTS, followed by pleasure (46.9%), peer pressure (22.8%), boredom (17.8%) and stress (10.8%). - 17.6% failed to identify a hazard with WTS use. - Majority deemed cigarettes as more harmful than WTS. - 30% of medical students regard WTS and cigarettes as equally harmful,, compared to 21.3% of non-medical students - 78.8% shisha pipe smokers perceived cigarettes to be more addictive than shisha. On the other hand, only 62.2% non-shisha pipe smokers considered cigarette smoking to have greater addictive properties. |
| **13.**  **Beliefs and attitudes related to narghile (waterpipe) smoking among university students in Syria.**  **Maziak et al.**  **2004** | - Sampling frame: Students at the University of Aleppo - Sampling Method: Convenient Sampling - Recruitment method: In person - Administration method:   In person, interview administered | - Sample size calculation: No - Sampling type: Non-probability sampling - Validity of tool: Self-developed, non-validated tool. Based on previously used tools on the assessment of cigarettes - Pilot testing: Yes - Response rate: 98.8% | - Country: Syria - Participants: 278 Males, 309 Females. Mean age: 21.8 years (SD: 2.1) - Setting: Student Dormitories. 2003 - N sampled: N/A - N participated: N/A - N analyzed: 587 | - ‘Trendiness’, alleviating boredom and shisha as a social activity were reasons for WTS in the current user population (n = 86), whereas adverse health effects and smoke and pollution were the main reasons given by non-smokers for disliking shisha (n = 433). - Students mainly associated WTS with respiratory illness. - They further believe WTS is more harmful than cigarettes. All students’ except one stated WTS is harmful for a foetus. - 89.5% those who utilised the shisha pipe perceived cigarettes to have increased addictive properties in comparison to shisha. On the other hand, 77.1% non-smokers believed cigarettes to have increased addictiveness when comparing to shisha smoking. |
| **14.**  **Fallacies about water pipe use in Turkish university students - what might be the consequences?**  **Alvur et al.**  **2014** | - Sampling frame:   Sakarya University campus   - Sampling Method: Simple Random Sample - Recruitment method: In person - Administration method: In person, self administered (under supervision of investigator) | - Sample size calculation: Yes - Sampling type: Probability Sampling - Validity of tool: Self developed tool, no validation reported - Pilot testing: Not reported - Response rate: Not reported | - Country: Turkey - Participants:   The mean±SD age of the students was 0.75±2.29years (min 18, max 32). There were 864 (68.8%) females and 391 (31.2%) males.   - Setting: Sakarya University – date not stated - N sampled: 1320 - N participated: N/A - N analyzed: 1255 | - 6.3% state that WTS is not harmful due to smoke not ‘burning the lungs’. 25.33% believe carcinogens are removed by the water in the shisha apparatus. 12.11% believe shisha has no nicotine. - Almost 1/5 students perceived shisha pipe with ‘fruit/aroma’ to not cause addiction. |
| **15.**  **Perceptions of Turkish University Students about the Effects of Water Pipe Smoking on Health.**  **Sahin et al.**  **2015** | - Sampling frame:   Students at Ankara University   - Sampling Method: Simple random sampling - Recruitment method:   In person   - Administration method:   In person, self-administered (under supervision of investigator) | - Sample size calculation: No - Sampling type: Probability sampling - Validity of tool: Part Self developed tool, not validated.   However also used previously reported validated tool.   - Pilot testing:Not reported - Response rate: Not reported | - Country: Turkey - Participants: 41.7% ≤ 19 years, 46.7% Male, 57.7% 1st year - Setting: Ankara University, September 2014 – January 2015 - N sampled: 907 - N participated: N/A - N analyzed: 877 | - Females and healthcare students’ generally have greater perception of the health effects of WTS. It was also noted students with higher ‘weekly pocket money’ had reduced perceptions as well as those who were current WTS users. Non-users had better perceptions and knowledge of WTS harms. - 52.6% of the study sample perceived shisha pipe smoking to have addictive properties. Likewise, 60.1% sample ‘strongly agreed’ with a statement claiming the act of shisha pipe smoking to have addictive properties. Similarly, 60.4% ‘strongly agreed’ with the fact that the flavoring of the shisha contributes to its addictive properties. |
| **16.**  **Argileh smoking among university students: a new tobacco epidemic.**  **Chaaya et al.**  **2004** | - Sampling frame: University Students - Sampling Method: Stratified Cluster - Recruitment method: In person - Administration method: In person self administered | - Sample size calculation: Yes - Sampling type: Probability sampling - Validity of tool: Self developed tool no validation reported - Pilot testing: Not reported - Response rate:100% | - Country: Beirut - Participants: 216 (52%) males, 200 (48%) females. Age 17-28 (19) - Setting: University - N sampled: 416 - N participated: 416 - N analyzed:416 | Answering the following questions:  1. Support banning WTS in the workplace:   - Smokers 87 (77%), Non-smokers 251 (91.9%)   2 Support banning WTS in public gardens smokers   - Smokers 40 (35.4%), Non-smokers 152 (55.5%)   3. Support banning WTS for those under 18 years   - Smokers 31 (27.9%), Non-smokers 31 (11.3%)   4. Support banning WTS in restaurants   - Smokers 16 (14.2%), Non-smokers 104 (37.8%)   5. Support banning WTS in commercials   - smokers 15 (13.4%), non-smokers 123 (44.2%)   Reasons for smoking argileh n=41:   - Entertaining 41 - Tasty 36 - Relaxing 30 - Time to think 12   When nonsmokers were asked  about their reaction if  in a closed room with WTS,:   - Do nothing: 23% - Distance themselves: 35.5% - Leave the room: 26% - Ask the smokers to stop (15%).   When waterpipe smokers were  asked about their reaction if present  in a closed room with nonsmokers:   - ~1/4 (26%) reported   they would continue smoking,   - 49% would stop smoking - 25% would leave the room.   The vast majority of smokers and nonsmokers supported banning WTS in the workplace.  Attitudes toward WTS varied across the faculties. 54% of students in the health sciences fields were generally negative about WTS compared to:   - 40% arts and sciences students, - 38% of engineering and architecture students - 31% of business students, - 27% among agriculture and   food sciences students.  Majority of respondents were aware  that WTS is related to respiratory  diseases (88%), heart diseases  (68%), fetal problems (62%), and  cancer of the oral cavity (63%).  A majority of respondents  recognized that WTS contains  addictive substances (67%),  poisonous gases (61%), and nicotine  (72%).  Lower percentages of respondents  knew that argileh contains  carcinogenic substances (50%), tar  (52%), and heavy metals (18%).  In addition, only a few students knew that argileh has been linked with infections (43%), ulcer (31%), disease transmission (45%), and increased carboxyhemoglobin in the blood (46%).  A large proportion of students also endorsed two popular misconceptions concerning WTS:   - The poisonous smoke concentration is reduced by water   filtration (77%) and by filtration at  the mouthpiece (76%). |
| **17.**  **Beliefs and Perceptions Toward Quitting Waterpipe**  **Smoking Among Café Waterpipe Tobacco Smokers**  **in Bahrain**  **Saif et al.**  **2013** | - Sampling frame: Shisha Cafes Bahrain - Sampling Method: Randomly selected Shsiha cafes - Recruitment method: In person - Administration method: In person. Self-administered | - Sample size calculation: Yes - Sampling type: Non-Probability - Validity of tool: Previously reported validated tool - Pilot testing: Not reported - Response rate:380 (90.7%) | - Country: Bahrain - Participants: Shisha smokers in café. Mean age 28.9. 92.4% males - Setting: Shisha cafes - N sampled: 400 - N participated: 380 - N analyzed: 380 | - Reasons for waterpipe smoking included boredom (58%), the social aspect of smoking (18%), and increased availability (12%). Most participants (73%) reported that their families did not support their use of waterpipe tobacco. - 43% of participants perceive waterpipe and cigarette smoking to be equally harmful to one’s health, with 29% thinking WTS is more hazardous than cigarettes and 28% that cigarettes are more hazardous than waterpipe. - 67% of participants regard cigarettes as more addictive than waterpipe. 81% of waterpipe smokers stated that they could quit smoking at any time, but only 40% were interested in quitting. The major reason for expressing an interest in quitting was personal or family health reasons (85%). The majority of those interested in quitting had a previous attempt to quit. - Many waterpipe smokers (55%) stated that they find it easy to stop smoking with only 8% finding it very difficult. Those with a university degree were more likely to think that they could quit waterpipe. |
| **18.**  **Cigarette and waterpipe smoking associated knowledge and behaviour among medical students in Lebanon.**  **Jradi et al.**  **2013** | - Sampling frame: Medical Students - Sampling Method: Convenience Sampling - Recruitment method: In person - Administration method: In person | - Sample size calculation: No - Sampling type: Non Probability - Validity of tool: Previously reported validated tool - Pilot testingNot reported: - Response rate: 191 54.3% | - Country: - Participants: Lebanon - Setting: University campus - N sampled: 354 - N participated: 191 - N analyzed:191 | - In response to the intention to quit waterpipe smoking question, 50.0% of the smokers reported that they intend to quit in the future. When asked about health risks associated with smoking, 42.8% of waterpipe smokers believed that waterpipe smoking was more harmful than cigarettes, whereas 46.0% of cigarette smokers replied that waterpipe smoking was more harmful than cigarette smoking. |
| **19.**  **Cigarettes and waterpipe smoking among medical students in Syria: a cross-sectional study.**  **Almerie et al.**  **2008** | - Sampling frame: University Students - Sampling Method: Stratified random - Recruitment method: In person - Administration method: In person self administered | - Sample size calculation: No - Sampling type: Probability Sampling - Validity of tool: Self developed tool, validation reported - Pilot testing: Not reported - Response rate: 570 (93.1%) | - Country: Syria - Participants: Male 340 Female 230 Mean age 20.5 - Setting: University - N sampled: 612 - N participated: 570 - N analyzed: 570 | Answering the following questions:   - Smoking should be banned in public places – Smokers 91.7% n=111, Nonsmokers 91.1% (368) - Will you advise patients in the future to quit smoking? SMOKERS Yes 34.2% n=41; Sometimes 49.1% n=59; No 16.7% n=20 NONSMOKERS Yes 30.3% n=122; Sometimes 51.2% n=206; No 18.5% n=74 - Do you think quitting smoking is difficult? SMOKERS Yes 56.7% n=68; NONSMOKERS Yes 51.9% n=202 - Do you think smoking waterpipe is religiously unacceptable? SMOKERS Yes 67.3% n=74; NONSMOKERS Yes 62.9% n=234 - Do you think a male smoker looks: SMOKERS More attractive 5.8% (7); Less attractive 52.1% (63); The same 9.1%(11) NON-SMOKERS More attractive 8.7% (35); Less attractive 51.4% (206); The same 39.9%(160) - Do you think a female smoker looks: SMOKERS More attractive 5.7% (7) Less attractive 85.2% (104) The same 9.1%(11); NON-SMOKERS More attractive 6.5% (26) Less attractive 79.2 (316) The same 14.3%(57) - What do you consider more harmful, cigarette or waterpipe? SMOKERS No difference 13.2% (14) Waterpipe is worse 76.4% (81) Cigarette is worse 9.4%(10) Waterpipe is not harmful 1.0% (1) NONSMOKERS No difference 17.3% (64) Waterpipe is worse 74.3% (274) Cigarette is worse 7.6%(28) Waterpipe is not harmful 0.8% (3) |
| **20. Hookah pipe smoking among health sciences students**  **N van der Merwe**  **2013** | - Sampling frame: University Students - Sampling Method: Convenient sampling - Recruitment method: Email and in person   Administration method: Internet and self administered | - Sample size calculation: Yes - Sampling type: Non probability - Validity of tool: Self developed no validation - Pilot testing:   Response rate: Not reported | - Country: South Africa - Participants: Make 36% Female 64%   Mean age 21.4   - Setting: University Campus - N sampled: 256 - N participated: 228 - N analyzed:228 | - The majority had a permissive attitude towards WTS pipe smoking, with 80% believed it to be socially acceptable, and 84% were willing to recommend it to others. - Contrary to this, the majority of individuals (74%) believed the practice should be subject to legal regulation. |
| **21.**  **Hookah smoking: characteristics, behavior and perceptions of youth smokers in pune, India.**  **Kakodkar et al.**  **2013** | - Sampling frame: Unknwn - Sampling Method: Snowball Sampling - Recruitment method:In person - Administration method: Self administered | - Sample size calculation: No - Sampling type: Non Probability - Validity of tool: Self developed tool validation reported - Pilot testing: none - Response rate: 100% | - Country: India - Participants: College students M 188 (67%) f 92 (38%) - Setting: Unknown - N sampled: 280 - N participated: 280 - N analyzed:280 | Students positive perceptions  regarding WTS:   - Sweet smell 138 (36.3%) - Relaxation 149 (39.2%) - Gives a kick 79 (20.7%)   Negative   - Pollution 148(43.4%) - Smoke Production 88 (25.8%) - Harmful to health 92 (26.9%) |
| **22.**  **Knowledge, attitude, and practice of water-pipe smoking among medical**  **students in Rawalpindi, Pakistan.**  **Haroon et al.**  **2014** | - Sampling frame: University students (Medical) - Sampling Method: Convenience sampling - Recruitment method: In Person - Administration method: In person, self administered | - Sample size calculation: No - Sampling type: Non-probability - Validity of tool: Self developed tool no validation - Pilot testing: None - Response rate:724 70.1% | - Country: Pakistan - Participants: University students (Medical) Male 219 30% Female 505 70% Mean age 20.6% - Setting: - N sampled: 1033 - N participated: 724 - N analyzed:724 | - Answering the following questions: - Shisha is dangerous for health - Nonsmokers 455 (93.8%), Waterpipe Smokers 117 (83.5%) - Shisha has significant amount of tobacco - Nonsmokers 378 (77.8%), Shisha Smokers 108 (77.1%) - Habitual Shisha smoking can cause peptic ulcer- Nonsmokers 126 (25.0%) Shisha Smokers 21 (15.4%) - Habitual Shisha smoking can cause lung cancer - Nonsmokers 391 (80.6%), Shisha Smokers 113 (80.7%) - Habitual Shisha smoking can cause Coronary Heart Disease - Nonsmokers 147 (30.3%) Shisha Smokers 38 (27.1%) - Habitual Shisha smoking can cause Diabetes Mellitus - Nonsmokers 6 (1.2%) Shisha Smokers 6 (4.3%) - Habitual Shisha smoking can cause communicable disease - Nonsmokers 51 (10.5%) Shisha Smokers 23 (16.4%) - Shisha is more dangerous to health compared to tobacco - Nonsmokers 193 (39.8%) Shisha Smokers 68 (48.6%) - Water Filters the smoke by removing toxic products - Nonsmokers 58 (12.0%) Shisha Smokers 47 (33.6%) - Burning Coal helps to burn cancer causing agents of tobacco - Nonsmokers 58 (12.0%) Shisha Smokers 26 (18.6%) - Fruity flavor has benefits - Nonsmokers 56 (11.6%) Shisha Smokers 45 (32.1%) |
| **23. Pattern and prevalence of smoking among students at King Faisal University, Al Hassa, Saudi Arabia**  **H.I. Al-**  **Mohamed**  **2010** | - Sampling frame: University Students - Sampling Method: Multistage proportionate - Recruitment method: In person - Administration method: In person self administered | - Sample size calculation: Yes - Sampling type: Probability - Validity of tool: Data were collected using an anonymous self-administered, modified Arabic version of the Global Youth Tobacco Survey questionnaire, with the addition of the modified Fagerström Test for Nicotine Dependence. - Pilot testing: None - Response rate:1382 | - Country: Saudi Arabia - Participants: University Students - Setting: University campus 100% males Mean age 21 - N sampled: 1382 - N participated: 1382 - N analyzed: 1382 | - Concerning the participants’ beliefs, there were significant differences between waterpipe smokers and non-smokers that smokers have more friends and that smoking should be banned in public places (P < 0.001). - There was no difference in the belief that it was easy to quit smoking (P = 0.061) - Only about 25% of participants in both groups thought it was not easy to quit. |
| **24. Perceived factors related to cigarette and waterpipe (ghelyan)**  **initiation and maintenance in university students of Iran**  **Roohafza et ak,**  **2011** | - Sampling frame: University Students - Sampling Method: Random - Recruitment method: Not reported - Administration method: In person interviewer administered | - Sample size calculation: Not reported - Sampling type: Non probability - Validity of tool: Not reported not done - Pilot testing: - Response rate: 95% | - Country: Iran - Participants: Male 170 Female 63 - Mean age 22 - Setting: University campus - N sampled: 812 - N participated: 95% - N analyzed: 233 | Reasons for initation and maintenance of WTS included:  Friend smokes WTS: 71.1% (Females), 95.2% (Males)  Father Smokes WTS: 44.2% (Females), 33.6% (Males  Achieving Social acceptability: 5.7% (Females), 19.2% (Males)  Becoming mature: 5.7% (Females), 9.6% (Males)  Being beloved: Females (8.2%), Males (19.2%)  Enjoyed the smell of the smoke: Female (96.5%), Males (57.7%). |
| **25.**  **Experimentation with and knowledge regarding water-pipe tobacco smoking among medical students at a major university in Brazil.**  **Martins et al.**  **2014** | - Sampling frame:   Medical students at the  Faculdade de Medicina  da Universidade de São  Paulo (2nd and 6th years)   - Sampling Method:   Convenience Sampling   - Recruitment method: Not reported - Administration method: In person, self administered | - Sample size calculation : No - Sampling type: Non-probability sampling   Validity of tool: Previously reported, validated tool   - Pilot testing:Not reported - Response rate: Not reported | - Country: Brazil - Participants:   Ages of the third-year and sixth year students were (mean) 22.0 ± 2.76 years and 24.0 ± 1.94 years, respectively. 36.4% of waterpipe smokers were female.   - Setting: 2nd semester at the University. 2008 – 2013. - N sampled: N/A - N participated: N/A - N analyzed: 586 | - More than 98% of the respondents knew that impurities in water-pipe tobacco smoke are not filtered out through the water bowl. |
| **26.**  **A web-based program to increase knowledge and reduce cigarette and nargila smoking among Arab university students in Israel: mixed-methods study to test acceptability.**  **Essa-Hadad et al.**  **2013** | - Sampling frame:   Arab university students in Israel   - Sampling Method: Convenience - Recruitment method: Mail/Internet - Administration method: Internet and In person, group discussion | - Sample size calculation: No - Sampling type: Non-probability sampling - Validity of tool: Self-developed, non validated tool - Pilot testing: Yes - Response rate: 63.2% | - Country: Israel - Participants:   Mean age was 25 years (SD 5). 68.9% female . More Muslim students that Christians—47.1% (106/225) versus 43.1% (97/225),  respectively. The majority (164/225, 72.9%) of students reported that they were religious.  73.3% undergraduate students. 70.2% (158/225) of students were single   - Setting: 2007 – 2010. Colleges and Universities in Israel. - N sampled: Not reported - N participated: 356 - N analyzed: 225 | - Students agreed that there is a real lack of awareness and knowledge regarding WTS. - Furthermore, the majority (40/56, 71%) agreed that WTS was socially and culturally acceptable. The following statements were made by participants:   “Before reading the feedback on nargila smoking, I had no idea how dangerous it was. Everyone knows  cigarettes are not healthy, but I didn’t realize that nargila (waterpipe) was just as bad as (cigarettes) or even worse. No  one has ever talked to me about health hazards associated with nargila smoking. To me, this is really scary since everyone today is smoking nargila. I know kids as young as 10 years who smoke nargila and their parents think it is ok. It’s socially acceptable, not like cigarettes. As a girl, I am not ashamed to smoke nargila  in public but I would never smoke cigarettes in public or in front of my parents and family”  “Nargila smoking just seems better for you than cigarette smoking. The tobacco is fruit flavored, the smell and taste of the smoke is very fruity. Also, it seems that with the water pipe, the water would clean out, or purify,the tobacco before you inhale it.” |
| **27.**  **Narghile (water pipe) smoking among university students in Jordan: prevalence, pattern and beliefs.**  **Dar-Odeh et al.**  **2010** | - Sampling frame: All University students at 3 Jordanian institutions - Sampling Method:   Convenient Sampling     - Recruitment method: In person - Administration method: In person, self-administered | - Sample size calculation: No - Sampling type: Non-probability - Validity of tool: Not Reported      - Pilot testing: Not reported - Response rate: Not reported | - Country: Jordan - Participants: 741 Males. Age range 16- 26 years. - Setting: University - N sampled: 1454 - N participated: 1454 - N analyzed: 1451 | - 143 students thought smoking watepipe is more harmful than cigarette smoking while 1165 thought it was not. - The most frequently stated harmful effects of waterpipe were: Respiratory diseases (540), cancer (503), cardiovascular, disease (291), and mouth disease (85). |
| **28.**  **A descriptive study of the perceptions and behaviors of waterpipe use by university students in the Western Cape, South Africa.**  **Daniels et al.**  **2013** | - Sampling frame: Students attending interdisciplinary modules - Sampling Method: Cluster random sampling - Recruitment method: In Person - Administration method: In person, self-administered questionnaire | - Sample size calculation: No - Sampling type: Probability Sampling - Validity of tool: Previously reported, validated tool - Pilot testing: Yes - Response rate: 60.2% | - Country: South Africa – Cape Town      - Participants: 64%   females and 139 (36%) males with a mean age of 22.2 (SD = 5.04) years. Approximately 50% of the sample identified themselves  as Colored (mixed  ethnicity) followed by  40% Black African, 6%  Whites and 4% Indians.   - Setting: College - N sampled: 415 - N participated: 250 - N analyzed: 250 | - Waterpipe users did not perceive the waterpipe to be a health risk. - Almost 50% of users, as compared to non-users, believed that a waterpipe has   less nicotine (43% and 17%, p<0.01), the dangers of smoking the waterpipe are exaggerated (48% and 13%, p<0.01), and the tobacco toxins are filtered by the water in the waterpipe (44% and 27%, p<0.01).   - The majority of users believed that smoking the waterpipe is not addictive (58% and 20%, p<0.01) and that users could quite easily quit (53% and 17%, p<0.01). - Furthermore, sharing the waterpipe was perceived as not harmful by waterpipe users (34% and 15%, p<0.01). - More non-users than users believed that long-term health problems such as heart disease (almost 50% and 32.51%, p<0.01), lung cancer (57.34% and 39.31%, p<0.01) and lung disease (57.33% and 39,36%, p<0.01) could develop. - The most common reason for smoking the waterpipe was for relaxation (67% and 65%) but this was not a significant finding, |
| **29.**  **Perception of young adults toward hookah use in Mumbai.**  **Dani et al.**  **2015** | - Sampling frame: Sample of students from south Mumbai college - Sampling Method: Convenience sample - Recruitment method: In person - Administration method: In person, self-administered | - Sample size calculation: No - Sampling type: Non-probability      - Validity of tool: Not reported - Pilot testing: No - Response rate: 89.4% | - Country: India - Mumbai - Participants:   62.6% were females,  with 73% nonusers  of waterpipe. 67% were  aged between 18 –  25.  About 27% were  waterpipe users comprising 50% males and 50% females.  Setting: College   - N sampled: 500 - N participated: N/A - N analyzed: 447 | - A Significant difference was observed in the perception of waterpipe being addictive in waterpipe users versus nonusers (P < 0.001). Furthermore, the users perceived waterpipe to be injurious to health (P = 0.001) and that it causes cancer (P < 0.05). - However, an equivalent percent of both users and nonusers perceived Waterpipe to be safer than cigarettes. This may be due to the infrequent use of waterpipe in a majority of our participants. Thus, it should be noted that educational messages need to be tailored to account for infrequent use. - Waterpipe users were more likely to have a close friend who smokes waterpipe (P < 0.001). Thus, educational messages demoralizing waterpipe use may not be effective in smokers, with waterpipe smoking in their peer groups. - On the other hand, significant difference in the perception that waterpipe is safer than cigarettes, presence of fruits in waterpipe, air quality of waterpipe parlor is harmful, ambience of the parlor, looking cool and enabling socializing, was not observed in the users and nonusers of waterpipe as well as in males and females. - A comparison between female and male waterpipe users indicated that 66.6% of females perceived watepipe as addictive while only 47.5% of males considered as addictive (P < 0.05). Both male (56.7%) and female (58.3%) users perceived waterpipe use as safer than cigarette smoking. The perception with reference to air quality of watepipe parlors being safer was equivalent in male and female users. - Whilst among the nonusers, both males and females indicated watepipe and cigarettes as equally harmful to the environment and the deteriorating air quality as not safe |
| **30.**  **Prevalence and Factors Associated with Sheesha Smoking in a Sample of Medical Students.**  **Babar et al.**  **2015** | Sampling frame: Students registered at medical Colleges in Rawalpindi, Pakistan (Foundation University Medical College, Islamic International Medical College, Yusra Medical College, Rawalpindi Medical College, Army Medical College)   - Sampling Method: Convenience Sampling - Recruitment method: In person - Administration method: Self administered | - Sample size calculation: Yes - Sampling type: Non-probability   Validity of tool:  Not Reported   - Pilot testing: Yes (states ‘pretested’ questionnaire) - Response rate: Not Reported | - Country: Pakistan - Rawalpindi   Participants:  Mean age was 21±2. 58% of study sample were females 231(40%) were from public sector medical colleges 347(60%) were from private sector medical col­leges. 165 (28.5%) of students in the study sample were 1st year medical students, 147(25.4%) were 2nd year 106(18.3%) were 3rd 102(17.8%) were 4th year58 (10%) were final year student.   - Setting: Medical college - N sampled: N/A - N participated: N/A - N analyzed: 578 | - 84 (40.5 %) said they started smoking waterpipe just for sake of fun, 56 (27%) said for sake of style, 39(19%) started just out of curiosity and 8(13.5) because of peer pressure. - 123 (59%) be­lieved it to be less harmful and 151(73%) believed it to be less addictive than cigarettes. 151 (73%) believed that WTS is socially more acceptable spe­cially for females. 139(67%) thought that WTS looked cool and attractive whereas 89 (43%) said that they feel craving for waterpipe - 107 (52%) believed that WTS increased their energy level where as 65 (31%) felt no change in the energy level. - 107(52%) wanted to quit WTS. - 151 (73%) believed that WTS is socially more ac­ceptable especially for females - 139 (67%) thought that WTS looked cool and attractive whereas 89(43%) said that they feel craving for waterpipe - 207(56%) do not want to smoke water pipe because they were aware of its harmful effects. - 255 (69%) said they have no future plans to start WTS in future. But 82(22%) were not sure about their future plan regarding WTS. |
| **31.**  **Psycho-social Needs Impact on Hookah Smoking Initiation among Women: A Qualitative Study from Iran.**  **Baheiraei et al.**  **2015** | - Sampling frame: Tehran women with previous waterpipe use - Sampling Method: Snowball Sampling - Recruitment method: N/A - Administration method: In person, interviewer administered. Self-administered questionnaire on waterpipe use also conducted | - Sample size calculation: No - Sampling type: Non-probability - Validity of tool: Self developed tool, no validation reported - Pilot testing: Yes - Response rate: N/A | - Country: Iran - Tehran   Participants:  Ages ranged from 15 to 51 years old. Median - 24 years. Age at onset of smoking watepipe ranged  from 7 to 42 years,  with a median age of  25 years.  Participants were  married, single or  divorced women  belonging to different geographic regions of Tehran and were from different ethnic subgroups  .  Most participants had  a diploma or  academic degree.   - Setting: Individual interviews not conducted in public places - N sampled: 49 - N participated: N/A - N analyzed: 36 | - A 23-year-old female university student who began smoking waterpipe at the age of 15 said: “We enjoyed little recreations. Tea houses were the best places for me and my cousins for this purpose. This was because recreations are scarce here, and the only cozy places for this purpose are tea houses. You cannot find such a cozy place in parks.” - Another motive for women was to keep others content or to join   them while they were smoking watepipe. A 23-year-old female university student who began smoking waterpipe at the age of 15 said: “When we go to a tea house together, we feel great and prestigious. When we go to a tea house together, we think that it's prestigious and that smoking waterpipe is part of our prestige… One feels great, you know. Grownups do this; so you like to say “hey, I have grown up, too”. |
| **32.**  **Smoking habits among medical students in Western Saudi Arabia.**  **Wali et al.**  **2011** | Sampling frame: All medical students at King Abdulaziz University,   - Sampling Method: N/A - Recruitment method: N/A - Administration method: N/A | - Sample size calculation: No - Sampling type: N/A - Validity of tool:   Self-developed, non-validated tool based on previously used tools (Global Tobacco Survey).   - Pilot testing: Not reported - Response rate: Not reported | - Country: Saudi Arabia – Jeddah - Participants:   69% Female. 46.8% < 22 years in age. 28.9% in 5th year (most prevalent year group). 2nd most prevalent year group was 2nd year students (20.7%)   - Setting: University - N sampled: N/A - N participated: N/A - N analyzed: 643 | - 9.5% thought that WTS was not harmful to health. - 1/3^rd^ (32.8%) of the students who thought water pipe is not harmful were actually water pipe smokers compared with 9.1% of those who thought it is harmful (p=0.001). |
| **33.**  **Students’ perspectives in Tehran University of Medical Sciences about factors affecting smoking hookah. Razi Journal of Medical Sciences.**  **Dehdari et al.**  **2012** | - Sampling frame: Unknown - Sampling Method: Convenience - Recruitment method: In person - Administration method: In person, self administered | - Sample size calculation: Unknown - Sampling type: Non-probability sampling - Validity of tool: Unknown - Pilot testing: Unknown - Response rate: Unknown | - Country: Iran - Participants: 162 Males - Setting: Tehran University - N sampled: N/A - N participated: N/A - N analyzed: 162 | - [Translated from Persian] - Reasons for smoking shisha include: cost-effectiveness, reduced anxiety, fatigue, access to café’s and having waterpipe friends |
| **34.**  **Waterpipe and Cigarette Smoking among**  **University Students in the Western Cape,**  **South Africa**  **Kruger et al.**  **2016** | - Sampling frame:   All registered students at publicly funded universities in the western cape.   - Sampling Method: Convenient - Recruitment method: Email - Administration method: Internet | - Sample size calculation: No - Sampling type: Non-probability sampling - Validity of tool: Previously validated tool - Pilot testing: Not Reported - Response rate: 5.7% | - Country: South Africa – Western Cape   Participants: 45.2% Female. With regards to ethnicity, 38.5% African, 25.4% mixed race and 31.7% white. 75.9% Christian.   - Setting: University - N sampled: 107,396 - N participated: 6111 - N analyzed: 4590 | - Students indicated that they smoke waterpipe to socialize (57.0%) or to relax (33.3%). |
| **35.**  **To assess the prevalence and factors associated with shisha smoking in medical students.**  **Asif et al.**  **2017** | - Sampling frame:   Not stated   - Sampling Method:   Method not stated – text only says ‘Simple Random Sampling’   - Recruitment method:   Not stated   - Administration method:   Not state | - Sample size calculation:   Not stated   - Sampling type:   Unknown (could be probability sampling as they stated they used simple random sampling)   - Validity of tool:   Self developed tool – no validity reported (but based on results from literature reviews)   - Pilot testing:   Not reported   - Response rate:   Not stated | - Country:   Pakistan   - Participants:   No demographics reported   - Setting: - N sampled: 578 - N participated: 578 - N analyzed: 578 | - 32.7% believed WTS is addictive, with 24% believing that it isn’t addictive - 25% want to quit WTS, 20% don’t know if they want to quit |
| **36.**  **Perceptions Of Health Professional Students Regarding Waterpipe Smoking And Its Effects On Oral Health.**  **Shuja et al.**  **2018** | - Sampling frame: University Students – Medical and Dental - Sampling Method:   Convenient sampling   - Recruitment method:   In person   - Administration method: - Questionnaire | - Sample size calculation: Yes - Sampling type: Non-Probability - Validity of tool:   Global Health Professions Student Survey – modified for waterpipe smoking   - Pilot testing: Not reported - Response rate: 100% | - Country: Pakistan - Participants: University Students - Mean age: 21.36 =/- 16.09   Male: n= 146 (42.7%)  Female: N=196 (57.3%)   - Setting:   University   - N sampled: 342 - N participated: 342 - N analyzed: 342 | - Two hundred and thirty-seven participants (69.3%) stated that waterpipe smoking had detrimental effects on health - In this study, most of the participants (69.3%) admitted that waterpipe smoking is more harmful than cigarette smoking for oral health. - One third of the respondents were unaware that WPS causes staining of teeth.   Serious Illness: YES 335(98%)  NO 7(2%)  Lung Disease YES 324(94.7%)  NO 18(5.2%)  Lung Cancer YES 320(93.3%)  NO 22(6.4%)  Oral Disease YES 311 (90.9%)  NO 31(9.1%)  Oral Cancer YES 307(87.9%)  NO 35(10.3%)  Stroke and Blood clot in the brain YES 223(65.2%)  NO 119(34.7%)  Stained Teeth YES 277(64.6%)  NO 115(33.6%)  Dental Caries YES 116(48%)  NO 176(51.5%)  Bad taste in Mouth YES 164(48%)  NO 178(52%)  Halitosis YES 178(52%)  NO 164(48%)  Effects Wound Healing YES 248(72.5%)  NO 94(27.5%)  Second hand smoke cause serious illness YES 226(66.1%)   - NO 116(33.9%) |
| **37.**  **Harm perceptions of waterpipe tobacco smoking among university students in five Eastern Mediterranean Region countries: A cross-sectional study.**  **Rmeileh et al.**  **2018** | - Sampling frame: University students - Sampling Method: Convenience Sampling - Recruitment method: Email - Administration method: Internet | - Sample size calculation: Yes - Sampling type: Non-Probability - Validity of tool: self developed tool, No validation reported - Pilot testing: - Not reported - Response rate: Does not state | - Country: Egypt, Jordan, Occupied Palestinian Territories, UAE and Oman - Participants: - Male 65.6% Female 34.4% - Setting: University - N sampled: ??? - N participated: 2544 - N analyzed: 2544 | - Overall, 72% of all students believed that waterpipe smoking during pregnancy can harm the baby, 73% believed that WTS can be harmful to children, 62% believed that WTS can cause fatal lung disease and 60% believed that quitting WTS can reduce serious risk to health. Around 50% of all students believed that WTS causes cancer, stroke and heart diseases. Less than 50% of the students believed WTS causes fatal lung disease for non-smokers (46%), can kill (47%) and is addictive (30%). The answers of students from Egypt indicated the lowest level of perceived WTS harm and their answers were significantly different from those of students from the other four countries |
| **38.**  **Waterpipe Smoking among University Students in Sulaimaniyah, Iraqi Kurdistan: Prevalence, Attitudes, and Associated Factors. Tanaffos.**  **Othman et al.**  **2017** | - Sampling frame: University campuses - Sampling Method: Multistage sampling - Recruitment method: Unclear - Administration method: Unclear | - Sample size calculation: Done with following parameters: reference population and estimated WPS prevalence of 10% - Sampling type: Probabiliy - Validity of tool: Self developed, not validated - Pilot testing: Not reported - Response rate: 91% | - Country: Iraq - Participants: University and college students   Male N=565 (53%)  Female N=496 (47%)   - Setting: University and college campuses - N sampled: 1160 - N participated: 1061 - N analyzed: 1061 | - Almost 67% of the participants said that waterpipe smoking was more harmful to health than cigarette smoking, and 33% said it was socially more acceptable than cigarettes. When nonwaterpipe smokers were asked whether they intended to start WPS, only 1.6% said that they intend to. - 89% of non-waterpipe smokers believed waterpipe smoking may cause addiction, only 62% of smokers believed so, and 70% of non-smokers vs. 57% of smokers believed that waterpipe smoking is more harmful to health than cigarettes. A total of 71% of smokers and only 8% of non-smokers said waterpipe smoking is “cool”; 63% of smokers and only 37% of nonsmokers believed that waterpipe smokers have more friends - Males showed more “favorable” attitudes towards WPS. For example, 75% of males vs. 88% of females believed WPS is addictive. - Siblings aware of behaviour N=194 (65.9%) - Parents aware of behaviour N=156 (53.3%) - Waterpipe smoking makes males more attractive – Males n=290/496(30.9%) Females n=142/565 (25.7%) - Waterpipe smoking females more attractive – Males n=77/496 (16.6%) Females n=40/565 (7.4%) - Waterpipe Smokers have more friends – Male n=237/496 (50.7%) Female 209/565(38.4%) - Waterpipe Smoking is part of our culture – Male n=56/496 (12.3%) Female n=84/565 (84%) |
| **39.**  **Water Pipe (shisha) Use and Legislation Awareness Against Shisha Smoking Among Medical Students: A study from Karachi, Pakistan.**  **Zavery et al.**  **2016** | - Sampling frame:   Not stated   - Sampling Method:   ‘Random convenient sampling’ (this is what they state, so I assume it was a convenient sample?)   - Recruitment method:   Not stated   - Administration method: Not stated | - Sample size calculation:   Yes, estimated sample size was 273 and the sample size obtained after inflating by a 10 % - estimated final sample size of 300 subjects.   - Sampling type:   Non-probability Sampling   - Validity of tool:   Self developed tool – after reviewing the literature and examining existing tools ?does this mean validity is reported?   - Pilot testing:   Not reported   - Response rate: 93.7% | - Country:   Pakistan   - Participants:   54.26 % were females  Mean age  21 ± 1.6     - Setting:   Aga Khan University (AKU) and Sindh Medical College (SMC)  October till December 2014   - N sampled: 450 - N participated: 422 - N analyzed: 422 | Peer pressure was the commonest reason to take up shisha (78 %). Some students cited glamour (8.7 %), few cited family members (5.49 %).  91.20% of those who used shisha believed that it was harmful; 6.5 % were unsure about the effects; only 2.19 % believed it was not harmful at all. 76.54% believed it contained tobacco.  74.64% students did not believe that the filtration system protects against the harmful effects of tobacco.  65.63% did not agree that shisha is less harmful that cigarettes.  63.74% did not know that there was legislation prohibiting SPS |
| **40.**  **Perception and practices of tobacco smoking among medical students in the Nile Delta, Egypt.**   - **Kabbash et al.** - **2018** | - Sampling frame:   Students participating in group practical tutorials   - Sampling Method:   Convenience Sampling   - Recruitment method:   Not reported   - Administration method: in person, self-administered | - Sample size calculation:   Yes, calculated at 10% but reasoning behind this value was not stated   - Sampling type:   Non-probability Sampling   - Validity of tool:   Self developed tool –no validity reported   - Pilot testing:   No   - Response rate: 83.8% | - Country:   Egypt   - Participants:   More than half were female (50.6%)  Ethnicity and age statistics not stated   - Setting:   4 faculties of medicine in the Nile Delta (Tanta, Mansoura, Menoufiya, and Zagazig)   - N sampled: 1715 - N participated: 1715 - N analyzed: 1438 | 63.3% disagree that shisha smoking is less hazardous than cigarettes, with a greater proportion of males (66.1%) disagreeing than females (60.6%) |
| **41.**  C**omparison of patterns of use, beliefs and attitudes related to waterpipe between beginning and established smokers**  **Asfar**   - 2018 | - Sampling frame:   Students in dormitories at the University of Alleppo   - Sampling Method:   Convenience Sampling   - Recruitment method:   In person   - Administration method:   In person, self administered | - Sample size calculation:   No   - Sampling type:   Non-probability Sampling   - Validity of tool:   Self developed tool –no validity reported   - Pilot testing:   No   - Response rate: Not Reported | - Country:   Syria   - Participants:   Male (82.6%). Majority Muslim (94.2%). 94.2% Single   - Setting:   University of Aleppo Dormitory   - N sampled: 587 - N participated:   Unknown   - N analyzed: - 86 | 89.% believe they can quite WTS anytime.  40.7% state they will quit WTS, and 65.7% attempted to quit last year.  Main motivation for quitting WTS is ‘no challenge’ (37.1%) and Friends (28.6%).  Main Health Risk of WTS is Respiratory Disease (47.9%). Overall >50% can identify health risks with WTS   - Cigarettes are more addictive than WTS. But WTS is more harmful. |
